# Psychosocial interventions for depression among young people in Sub-Saharan Africa: a systematic review and meta-analysis

**DOI:** 10.1186/s13033-024-00642-w

**Published:** 2024-06-22

**Authors:** Lotenna Olisaeloka, Echezona Udokanma, Asma Ashraf

**Affiliations:** 1 Institute for Global Health, University College London, London, UK; 2 School of Nursing, Midwifery, and Health, Coventry University, Coventry, UK; 3 Department of Nursing, City University of London, London, UK

**Keywords:** Depression, Mental health, Adolescents, Youth, Young people, Psychosocial interventions, Psychological therapy, Cognitive behavioral therapy, Interpersonal psychotherapy, Sub, Saharan Africa (SSA), Low- and Middle-Income Countries

## Abstract

**Background:**

Depression among young people is a global health problem due to its rising prevalence and negative physical and social outcomes. The prevalence of depression and the treatment gap among young people in Sub-Saharan Africa (SSA) is higher than global estimates. Most psychosocial interventions for adolescent and youth depression were developed in high-income countries and less is known about their effectiveness in SSA. Due to contextual differences, findings from High-Income Countries (HICs) are less applicable to SSA. Yet, no systematic review of psychosocial interventions for depression among young people in SSA has been conducted.

**Methods:**

A systematic literature search of four databases (Medline, Web of Science, PsycInfo, and Cochrane library) was conducted. Experimental studies published before May 2024 that evaluated the effect of psychosocial interventions on depressive symptoms among young people (aged 10–24 years) in SSA were included in the systematic review. Effect sizes (Hedge’s g (g)) indicating differences between intervention and control groups were calculated using a random effects model.

**Results:**

Twenty-two eligible studies were identified for the systematic review, of which eighteen randomized control trials (RCTs) involving 2338 participants were included in the meta-analysis. The findings revealed that psychosocial interventions significantly reduced depressive symptoms (g = −1.55, 95% CI −2.48, −0.63), although heterogeneity was high (I^2^ = 98.8%). Subgroup analysis revealed that efficacy differed significantly by intervention type, with Cognitive Behavioural Therapy (9 studies) showing the strongest effect (g = −2.84, 95% CI −4.29; −1.38). While Wise Interventions (a form of positive psychology interventions; 2 studies) had a moderate effect (g = −0.46, 95% C.I −0.53, −0.39), Interpersonal Psychotherapy (2 studies; g = −0.08, 95% CI −1.05, 0.88) and Creative Psychological Interventions (3 studies; g = −0.29, 95% CI −1.38, 0.79) showed smaller, non-significant effects. Sensitivity analysis excluding studies at high risk of bias strengthened the effect size. Few studies assessed factors affecting intervention efficacy and showed mixed effects of age, gender, and adherence levels.

**Conclusion:**

Psychosocial interventions, particularly CBT, significantly reduced depressive symptoms among young people in SSA. However, it is crucial to acknowledge the high heterogeneity which likely stems from variations in study populations and intervention delivery modalities. This highlights the need for further research to identify the specific intervention components and delivery methods that work best for distinct subpopulations. Future research should also explore how long intervention effects are maintained and factors affecting efficacy.

**Supplementary Information:**

The online version contains supplementary material available at 10.1186/s13033-024-00642-w.

## Background

### Depression is a global health issue

Depression is a common mental disorder that affects about 280 million people globally and is the second leading cause of disability worldwide [1, 2]. Depression is a mood disorder characterized by a persistent feeling of sadness and loss of interest accompanied by somatic and cognitive changes that affect an individual’s ability to function [3]. The lifetime prevalence of depression varies by sex and region, ranging from 2.6% among males in the World Health Organization (WHO) Western Pacific Region to 5.9% among females in the WHO African Region [Sub-Saharan Africa (SSA)] [4]. The forty-eight African countries that lie south of the Sahara make up SSA. It is the poorest region in the world and contains twenty-four of the twenty-seven countries in the World Bank’s Low-income classification [5]. Although the estimated prevalence of depression in SSA is considerably high, the true prevalence is possibly higher due to underdiagnosis caused by stigma, paucity of mental health services, and inadequate research [6]. About 75–90% of people with depression and other mental disorders in low-and-middle-income countries (LMICs) do not receive treatment [7, 8]. This “treatment gap” is higher in many SSA countries. For instance, it is 99.8% in Sierra Leone [9]. Lack of treatment despite increasing prevalence results not only in disability but also productivity losses, which cost the global economy $1trillion annually [10].

### Depression among young people

The term "young people" refers to adolescents and youths. Adolescents are young people aged 10 to 19, while youths are between ages 15 and 24 [11]. Depression is the second most prevalent mental disorder among young people [2] and warrants increased attention for the following reasons. First, depression usually starts during adolescence and persists into adulthood, especially when undiagnosed or inadequately treated. Research shows that 50% of Common Mental Disorders (CMDs) appear by age 14 and three-quarters by age 24 [12]. The prevalence of depression is low in children (1%), then rises substantially during adolescence, especially among females [13]. This observed increase is attributable to the sociobiological changes typical of the post-pubertal phase, such as increased social understanding and self-awareness, changes to the brain circuits involved in responses to reward and danger, and elevated stress [14, 15]. Secondly, the prevalence of adolescent depression is rising. A recent meta-analysis revealed that the global point prevalence of depression in young people (25.2%) had doubled from pre-pandemic estimates [16]. The pandemic exacerbated an already rising prevalence observed in both High Income Countries (HICs) and LMICs over the last two decades [17–20]. Currently, the point prevalence of adolescent depression in LMICs ranges from 18% in China to 51% in Zambia [21]. Thirdly, the negative consequences of depression in young people are enormous. Adolescent depression is a major risk factor for suicide, which is a leading cause of death among young people, particularly in LMICs [22]. Furthermore, depression is associated with self-harm, substance use, risky sexual behaviour, and poor educational attainment [23, 24]. Although prevention, early diagnosis, and treatment can reduce its high burden and negative outcomes, 80% of young people with CMDs in LMICs and almost 100% in many SSA countries do not get the care they need [25].

### Depression among young people in Sub-Saharan Africa

Young people account for a third of SSA’s population [26]. One in ten young people in SSA suffers from CMDs, particularly anxiety and depression [27]. A recent systematic review estimated the point prevalence of depression among young people in SSA at 26.7% and shows that the prevalence in many SSA countries is higher than the global estimate [28]. This is likely due to contextual risk factors such as poverty, conflict, poor healthcare, HIV, and teenage pregnancy, in addition to those common in HICs (e.g., parental psychopathology) [27]. Despite this, only nine of the forty-eight countries in SSA have comprehensive policies for adolescent mental health, resulting in huge barriers to care [29]. It is on this premise that WHO, in its landmark World Mental Health Report, calls for contextually appropriate, cost-effective interventions for adolescent depression in SSA [22]. For every $1 invested in these interventions, Stelmach et al*.* expect $125 in health and economic benefits returned to the regional economy [25].

### Psychosocial interventions for depression in young people

Psychosocial Interventions for mental disorders are interpersonal or informational activities/techniques that influence outcome through changes in mediating biopsychosocial factors. They include psychological therapies like Cognitive Behavioural Therapy (CBT), Interpersonal Psychotherapy (IPT) and Psychodynamic therapy, and social interventions like peer support services and skill building [30]. Psychosocial interventions are the first-line approach for depression in young people and antidepressants should be used only in cases unresponsive to psychological therapy [31, 32]. Research over the years have established the efficacy of psychosocial interventions in the treatment of adult depression [33–35]. Given this evidence, different interventions have been adapted for adolescent and youth populations. CBT and IPT are the most extensively tested in young people and reviews have shown that they reduce depressive symptoms [36–38]. Attachment-based family therapy, though less extensively researched, has also shown some positive effect [39]. These interventions have proven effective when delivered in individual and group formats [40, 41] and via bibliotherapy or technology-assisted methods, although to varying degrees [42, 43]. They have also been delivered in different settings, such as schools and communities [38].

Majority of the studies that established these interventions as evidence-based were conducted in HICs and less is known about their effectiveness in LMICs. In recent years, these interventions have increasingly been tested in LMICs. Findings show that interventions developed in HICs might not be acceptable, feasible, or effective in LMICs due to contextual differences such as dissimilar cultural perceptions of depression, and barriers to care (e.g., low awareness, insufficient mental health workers, stigma, and poverty) [44]. Innovative solutions like task-shifting (use of non-mental health professionals), cultural adaptation, and the use of digital technologies have been tested with mixed results [45, 46]. There is a need to understand how these interventions are adapted to fit different contexts and how these modifications affect their effectiveness. Systematic reviews of LMICs involve only a few SSA countries, thus limiting their applicability to the region [44, 47, 48]. Though it remains unclear which psychosocial interventions are most effective in the region, no systematic review has been conducted on this topic. This review aims to identify and describe psychosocial interventions for depression among young people in SSA, determine their efficacy and explore factors that affect their efficacy. As most SSA countries do not have policies for young people’s mental health, findings from this study will contribute to future research and policy development.

## Methods

This systematic review followed the Preferred Reporting Items for Systematic Reviews and Meta-Analyses (PRISMA) guidelines [49] [Additional Files 1, 2].

### Search strategy

The search strategy was developed with the assistance of a research librarian and the systematic database search was first conducted in July 2022 (updated May 2024) with keywords identified using the PICO framework [50] (Table 1). The keywords with their MeSH terms and synonyms were combined with Boolean operators (“AND” and “OR”) and wildcards (*, ?) to run a comprehensive search on Medline (OVID). This search strategy was then adapted to Web of Science, PsycInfo and Cochrane Central Register of Controlled Trials (CENTRAL). The detailed search strategy for each database is shown in Additional File 3. The reference lists of included articles were also searched to identify other relevant papers.

**Table 1:** Identification of search terms using PICO framework

PICO component	Keywords	MeSH term and synonyms
Population	Young people Sub-Saharan Africa	MeSH: adolescent Synonyms: Adolescent* OR Teenager* OR Teen* OR youth* OR young person OR young people OR youngster* OR young adult* OR student* OR high school OR college OR secondary school MeSH: Africa South of the Sahara Synonyms: Sub-Saharan Africa OR SSA OR West* Africa OR East* Africa OR South* Africa OR Central Africa OR ( +)
Intervention	Psychosocial Intervention	MeSH: psychotherapy Synonyms: psychosocial OR psychological OR psychoeducation OR behavioural therapy OR cognitive behavioural therapy OR CBT OR Interpersonal Psychotherapy OR IPT OR problem-solving therapy OR counselling OR narrative therapy Synonyms: Treatment OR Therapy OR Trial OR Project OR Program*
Outcome	Depression	MeSH: depression; depressive disorder Synonyms: depression or depressive disorder* or dysthymia or depressive symptom

( +) – List of all Sub-Saharan African countries with “OR” between them

### Eligibility criteria

The inclusion and exclusion criteria were generated using the PICOS framework [50], as shown in Table 2. Studies that reported the effect of various psychosocial interventions on depressive symptoms in any adolescent or youth population in SSA were included. To best capture the state and quality of research, papers were not included or excluded based on study design or quality assessment. Due to limited resources for translation, only papers in English language were included.

**Table 2:** Inclusion and exclusion criteria

PICO component	Inclusion criteria	Exclusion criteria
Population	• Study population including young people (WHO definition: 10–24 years) • Young people (adolescents and youth) in SSA	• Primarily children or adult population • Study population outside 10–24 years • Young people living in non SSA countries
Intervention	• Studies on psychosocial interventions for depression • Intervention aimed at reducing depressive symptoms	• No intervention or pharmacological and other types of interventions • Intervention not focused on reducing depressive symptoms
Comparison	• All types of control arms • Quasi-experimental studies without control groups	• No exclusion based on use or characteristics of control group
Outcome	• Study reports outcome on depressive symptoms • Standardized instruments used to diagnose elevated depressive symptoms	• Study does not report outcome on depressive symptoms • Standardized instruments not used
Study design	• Primary experimental studies using different study designs (not limited to RCTs)	• Systematic reviews and meta-analysis
Others	• Studies reported in English language • Studies published in peer-reviewed journals	• Studies reported in other languages • Not peer-reviewed papers, grey literature¸ non-academic papers (commentaries, editorials, etc.), books, book chapters

### Data management and extraction

All records captured by the search terms were exported to EndNote 20 Library. After de-duplication, titles and abstracts were independently screened by two reviewers (LO and EU). Papers that did not meet the eligibility criteria were excluded**.** The full texts of the remaining papers were screened by same reviewers against the eligibility criteria. A data extraction form was developed to extract relevant information from the papers such as country, study design, intervention setting, screening instrument, intervention characteristics, and outcome (depressive symptoms pre- and post-intervention). Three tables were developed from this form and are presented in the results section.

### Risk of bias assessment

The Cochrane Risk of Bias tools for randomized control trials (RCTs) version 2 (RoB2) and Risk of Bias in Non-randomized Studies of Interventions (ROBINS-I) were used to assess the risk of bias (RoB) for RCTs and Non-Randomized Studies of Interventions (NRSI) respectively [50]. These specific RoB tools were used due to the methodological differences between study designs, particularly randomization which is an important consideration in judging bias. For RCTs, six RoB domains (randomization process, deviation from intended intervention, missing outcome data, measurement of outcome, and selective reporting) were assessed and studies judged to have high, “some concerns” or low RoB. For NRSI, six domains (confounding, participant selection, deviation from intervention, missing data, outcome measurement, and selective reporting) were assessed. Studies were judged as having a low, moderate, serious, or critical risk of bias [50].

### Meta-analysis

The meta-analysis included clinical trials wherein participants were randomly allocated to either receive a psychosocial intervention or be placed in control conditions, with depression scores reported as an outcome. Statistical analysis was performed using R software (version 4.3.1) and the *metafor* package (version 4.4.0). To ensure uniformity and reproducibility of results, standardized mean differences (SMD) alongside 95% Confidence Intervals (CI) were calculated for each study using extracted data (mean and standard deviation). SMDs were employed because studies used different screening instruments to evaluate depression scores [50]. Pre- to post-intervention changes were analysed but follow-up impacts were not considered due to a lack of information in some studies and variations in follow-up periods. Effect sizes (SMD) were calculated using Hedges’ g because it corrects for small sample bias. This was pertinent given the relatively small sample sizes in some included studies [51]. Effect size magnitudes were categorized as small (0.20–0.50), moderate (0.50–0.80), and large (> 0.80) based on Cohen's rule of thumb [52]. A random-effects model was used a priori to account for expected heterogeneity among studies, including variations in intervention types, delivery modalities, participant characteristics, and screening instruments. Statistical heterogeneity was assessed using Cochran’s Q test and I^2^. An I^2^ value of 0 to 25% can be considered as low, 50% as moderate and 75% and above as a high level of heterogeneity. Subgroup analysis was pre-determined to explore variations in psychosocial interventions [52]. Sensitivity analysis was conducted by excluding studies with high risk of bias to assess the robustness of findings. Publication bias was evaluated using a funnel plot and Egger’s regression test [53].

## Results

**Fig. 1:**
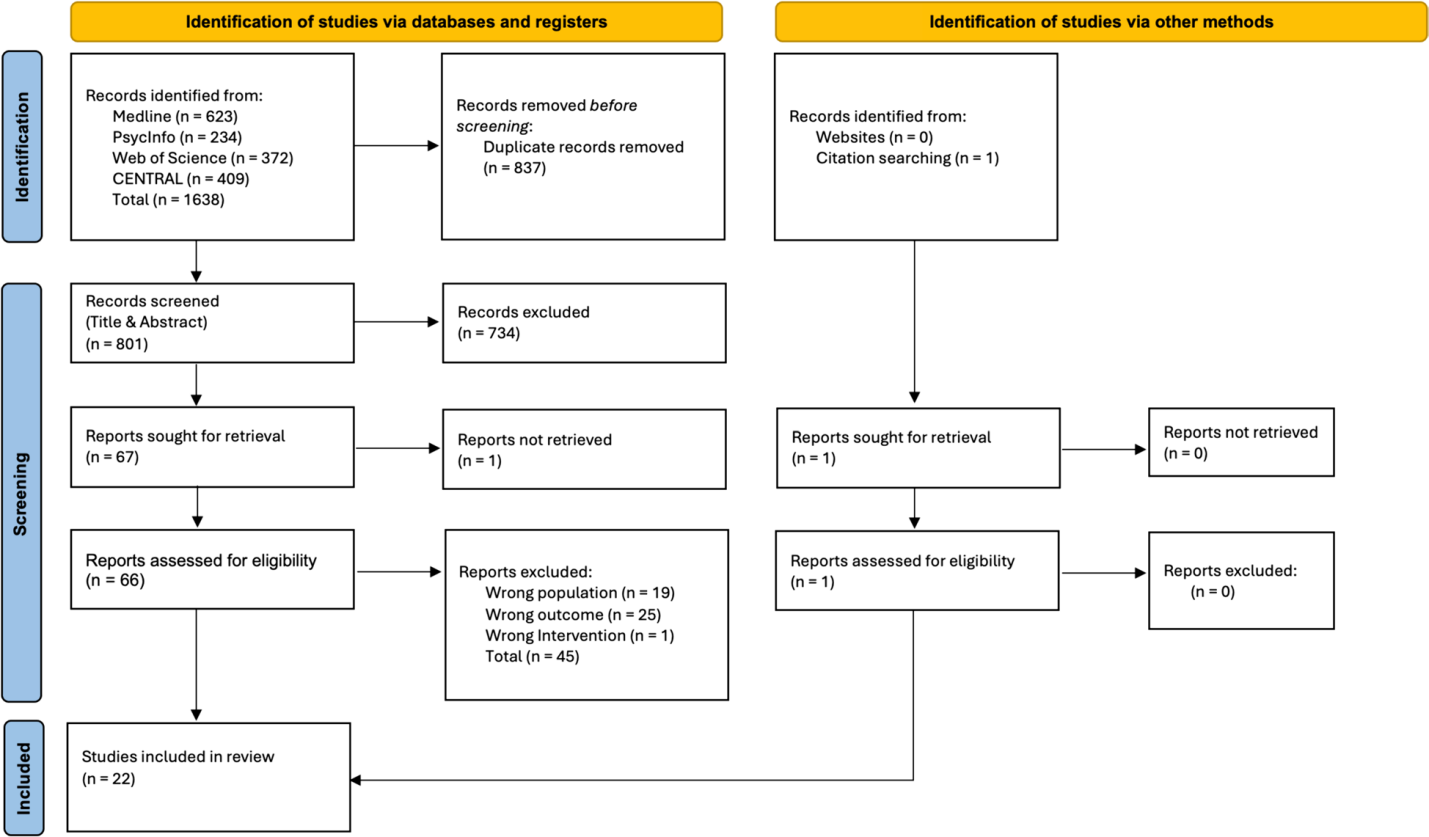
PRISMA flow diagram illustrating steps to paper selection

The search strategy identified 1,638 papers across the four databases. After de-duplication and title and abstract screening, 67 papers were sought for full-text screening. One could not be retrieved despite attempts to contact the author. Forty-five papers were excluded after full-text screening and one paper was identified via citation searching. Reasons for exclusion are listed in Additional File 4. Hence, a total of 22 studies were included in the review. The PRISMA Diagram illustrates the selection process (Fig. 1).

### Description of included studies

The twenty-two included studies were conducted across ten different SSA countries; nine in Nigeria [54–62], four in Kenya [63–66], two each in the Democratic Republic of Congo (DRC) [67, 68] and South Africa [69, 70] and one each in Botswana [71], Burundi [72], Mauritius [73], Rwanda [74], and Uganda [75]. Ninteen studies were RCTs, while three were NRSIs. Among the NRSI, one was a controlled clinical trial (CCT) [55] while two were pre-post intervention studies [54, 61]. Seventeen studies included only adolescents while the other six included youths. Table 3 shows the important characteristics of all included studies. The studies were carried out in different settings and targeted different populations. Most studies (fourteen) were conducted in schools; nine in secondary schools, and six in tertiary institutions. While the majority of interventions included the general student population, others targeted specific populations with increased risk of depression such as students with sickle cell disease in Nigeria (SCD) [54], internally displaced [63] and students from low-income families in Kenya [64–66], and orphaned students in Rwanda [74]. Among the non-school-based interventions, three were delivered in clinics to adolescents living with HIV in Botswana [71], trauma-exposed adolescents in South Africa [70] and depressed adolescents attending a psychiatric clinic in Nigeria [61]. The others were delivered in communities to war-affected adolescents in DRC [67, 68, 75] and orphans of the HIV epidemic in South Africa [69]. Some studies excluded participants with co-morbid psychiatric disorders, substance use, intellectual difficulties, and suicidality [55, 56, 60, 62, 68, 74]. Others also excluded participants based on depression screening. For example, while Are et al. [55] excluded people with severe depression, Eseadi et al. [58] included only individuals with moderate-to-severe depression. In contrast, three studies did not apply these exclusion criteria [64, 65, 67]. McMullen et al. noted that this was done to “keep the trial as naturalistic as possible” [67]. Overall, eleven studies had relatively small sample sizes (less than 60).

**Table 3:** Description of included studies

References	Study location	Study design	Study setting	Sample population	Sample Size	Screening instrument	Length of follow up
Adegbolagun et al. [54]	Nigeria	Quasi-experimental (Pre-Post Intervention)	University Health Centre	Students with sickle cell disease aged 16–24 years	20	Hospital Anxiety and Depression Scale (HADS)	None
Are et al. [55]	Nigeria	Controlled clinical trial	Secondary School	Students aged 13–18 years with clinically diagnosed depression	40	Beck's Depression Inventory (BDI)	None
Bella-Awusah et al. [56]	Nigeria	Randomized control trial	Secondary School	Students aged 14–17 years	40	Beck's Depression Inventory	16 weeks
Bolton et al. [75]	Uganda	Randomized control trial	Internally Displaced People (IDP) camps	Adolescent survivors of war and displacement aged 14–17 years	314	Acholi Psychosocial Assessment Instrument (APAI)	None
Ede et al. [57]	Nigeria	Randomized control trial	College of Education students	College students aged 15–25 years	162	Centre for Epidemiological Studies Depression Scale for Children (CES-DC)	12 weeks
Eseadi et al. [58]	Nigeria	Randomized control trial	University Campus	University students	67	Beck's Depression Inventory	2 weeks
Ezegbe et al. [59]	Nigeria	Randomized control trial	University Campus	Undergraduate social science students	55	Goldberg Depression Scale (GDS)	12 weeks
Ezeudu et al. [60]	Nigeria	Randomized control trial	University Campus	Undergraduate chemistry students	23	Beck's Depression Inventory	Not reported
Getanda and Vostanis [63]	Kenya	Randomized CONTROL TRIAL	Secondary School	Internally Displaced adolescents aged 14–17 years	54	Depression Self-Rating Scale for Children (DSRS)	1 week
Isa et al. [61]	Nigeria	Quasi-experimental (Pre-Post Intervention)	Psychiatric hospital	Clinically depressed adolescents aged 13–18 years	18	Beck Depression Inventory (BDI), Short Mood and Feelings Questionnaire (SMFQ)	4 weeks
Kaminer et al. [70]	South Africa	Randomized control trial	University-based trauma research clinic	Trauma-Exposed Adolescents aged 11–19 years	75	Beck Depression Inventory (BDI)	12 weeks
McMullen et al. [67]	Democratic Republic of Congo (DRC)	Randomized control trial	Re-Orientation Centre for former child soldiers	Former boy soldiers aged 13–17 years	50	African Youth Psychosocial Assessment Instrument (AYPA) (formerly known as the Acholi Psychosocial Assessment Instrument)	12 weeks
O'Callaghan et al. [68]	DRC	Randomized control trial	Local vocational training centre	War-affected sexually abused girls aged 12–17 years	52	African Youth Psychosocial Assessment Instrument (AYPA)	12 weeks
Ofoegbu et al. [62]	Nigeria	Randomized control trial	University Campus	University students	192	Beck's Depression Inventory	4 weeks
Olashore et al. [71]	Botswana	Randomized control trial	HIV Clinic	Adolescents living with HIV, aged 15 to 19	50	Patient Health Questionnaire-8 (PHQ-9)	24 weeks
Osborn et al. [65]	Kenya	Randomized control trial	Secondary School for low-income students	Secondary school students aged 13–18 years	103	Patient Health Questionnaire-8 (PHQ-8)	2 weeks
Osborn et al. [64]	Kenya	Randomized control trial	Secondary School in an urban slum	Secondary school students aged 12–19 years	51	Patient Health Questionnaire-8	None
Osborn et al. [66]	Kenya	Randomized control trial	High schools in an impoverished urban neighbourhood	Grade 9–11 High school students aged 13–19 years	235	Patient Health Questionnaire–8	4 weeks
Rivet-Duval et al. [73]	Mauritius	Randomized control trial	Secondary School	Secondary school students aged 12–16 years	160	Reynolds Adolescent Depression Scale—2 (RADS-2)	24 weeks
Thurman et al. [69]	South Africa	Randomized control trial	Community-based program	HIV orphaned and vulnerable adolescents 14–17 years	489	Centre for Epidemiologic Studies Depression Scale for Children (CES-DC)	48 weeks
Tol et al. [72]	Burundi	Cluster randomized control trial	Secondary school in war-torn area	War-affected secondary school students	329	Depression Self-Rating Scale for Children (DSRS)	12 weeks
Unterhitzenberger and Rosner [74]	Rwanda	Randomized control trial	Orphanage's boarding school	Orphaned adolescents aged 14–18 years	69	Mini International Neuropsychiatric Interview for Children and Adolescents, Part A, on depression (MINI-KID A)	None

Nine different screening instruments were used to assess depressive symptoms, with Beck’s Depression Inventory (BDI) being the most used. All the instruments, except the Acholi Psychosocial Assessment Instrument (APAI), were developed in western countries. APAI was developed and used in Uganda by Bolton et al. [75]. It was modified into the AYPAI and used in the two DRC studies. The reliability of APAI was 84%, similar to BDI, while AYPAI was 74% [67, 68].

All but five studies [54, 55, 65, 74, 75] reported mean depressive symptoms after a follow-up period (Table 3). The majority of studies adopted a three-month follow-up period [57, 59, 67, 68, 72]. The longest follow-up period was twelve months [69] while the shortest was 1 week [63]. One study had no follow-up [65] while another [60] did not report the length of follow-up.

### Intervention characteristics

The majority of studies (13) tested the effect of CBT-based interventions on depressive symptoms [54–62, 67, 68, 70, 72]. Two studies each exammined the effects of Interpersonal Psychotherapy (IPT) [69, 75] and Wise Interventions (WI) [64, 65]. Three studies tested Creative Psychological Interventions (CPI) such as Expressive Writing (EW) [63, 74] and arts-based therapy [66]. The other interventions combined two psychotherapautic approaches. The Resourceful Adolescent Program (RAP), a universal preventive programme, combined both IPT and CBT techniques [73] while the intervention by Olashore et al. [71] combined psychoeducation and problem-solving. Table 4 shows the important intervention characteristics of each study.

**Table 4:** Intervention characteristics

References	Intervention (Number of participants)	Control (Number of participants)	Intervention description/details	Frequency and duration	Delivery format and modality	Delivery personnel/facilitators	Cultural and contextual adaptation
Adegbolagun et al. [54]	Group CBT (18)	NA	A manualized CBT intervention with sessions on management of negative thoughts and feelings, activity scheduling, relaxation techniques, and attention diversion. Content adapted to reflect realities of living with Sickle Cell Disease	Weekly sessions for 5 weeks	Group Face to Face	Primary care physician with postgraduate training in adolescent mental health	Unspecified content adaptation to suit local culture. Sessions scheduled on weekends only to suit student’s academic schedule. Sessions condensed from 12 to 5 to enhance feasibility
Are et al. [55]	Group CBT (20)	Waitlist (20)	A manualized CBT intervention focused on psychoeducation, behavioural activation, activity scheduling, and relaxation strategies	1 h weekly sessions for 5 weeks	Group Face to Face	Teachers trained and supervised by a psychiatrist	Manual adapted to include local metaphors and exemplars. Encouragement of helpful pre-existing cultural and religious coping strategies used by both Christians and Muslims in Nigeria. Manual translated to local language (Hausa). Intervention delivered during school hours to encourage adherence
Bella-Awusah et al. [56]	Group CBT (20)	Waitlist (20)	A manualized CBT program with sessions on psychoeducation, behavioural activation, activity scheduling and relaxation techniques	45–60 min weekly sessions for 5 weeks	Group Face to Face	Consultant Psychiatrist with training in CBT	Manual included religious-based coping strategies and cultural analogies used in the local population. Sessions delivered in both English and local language (Yoruba)
Bolton et al. [75]	Group IPT (105)	Creative Play (105), Waitlist (104)	A manualized IPT intervention focused on identifying and dealing with interpersonal triggers and group relationship building	1.5-2 h weekly sessions for 16 weeks	Group Face to Face	Lay facilitators trained and supervised by mental health experts	Adapted to local culture (Acholi). Use of locally developed screening instrument (APAI) which reflects cultural perception of depression
Ede et al. [57]	Group CBT (82)	Waitlist (80)	A manualized CBT intervention which focused on cognitive restructuring (identifying, challenging, and modifying maladaptive schematic beliefs associated with depression)	1 h weekly sessions for 12 weeks	Group Face to Face	Experienced adolescent mental health counsellors	Sessions delivered in English and local language (Igbo). Manual otherwise not adapted
Eseadi et al. [58]	Rational Emotive Behavioural Therapy (REBT) (34)	Waitlist (33)	Religious REBT techniques, in addition to general REBT techniques for treatment of depression, were used. Incorporated scriptural contents and religious resources relevant to the students' religious traditions and orientations	2-h weekly sessions of 12 weeks	Group Face-to-face	NR	Adapted to incorporate religious philosophies and traditions of the undergraduate religious education students in Southern Nigeria
Ezegbe et al. [59]	Group CBT (28)	Waitlist (27)	CBT manual addressed mood monitoring techniques, cognitive restructuring, relaxation, reframing and problem-solving techniques	2 h weekly sessions for 12 weeks	Group Face to Face	Professional therapists	None
Ezeudu et al. [60]	Rational Emotive Behavioural Therapy (REBT) (12)	Usual care (11)	Group REBT Depression Manual used cognitive, behavioural, and emotive techniques to assist patients in identifying and altering irrational and self-defeating thoughts and beliefs which result in depression	Weekly sessions for 12 weeks	Group Face to Face	NR	None
Getanda and Vostanis [63]	Writing for Recovery (WfR) (27)	Waitlist (27)	A psycho-social-educational structured writing intervention which adopted a testimonial/narrative approach	Six sessions over three days	Group Face to Face	Paraprofessionals (with social care background)	Involvement of community stakeholders like schoolteachers, parents and religious leaders in intervention planning and implementation. Delivery of intervention in local Swahili language
Isa et al. [61]	Group CBT (18)	NA	A group-based manualised intervention focused on psychoeducation and basic CBT strategies (positive self-talk and behavioural activation)	30–40 min weekly sessions for four weeks	Group. Face to Face	Psychiatrist	Used manual developed by Nigerian experts to suit the local context Sessions delivered in commonly used local language (simplified Pidgin English)
Kaminer et al. [70]	Trauma-focused CBT (37)	Treatment as Usual (38)	An abbreviated 8-session version of TF-CBT, retaining all components of the original 12–15 session model but condensed into fewer sessions. Components included psychoeducation, relaxation skills, affective modulation, cognitive coping skills, trauma narrative and cognitive processing, enhancing safety, and caregiver sessions focused on parenting skills	90 min sessions weekly sessions for eight weeks	Individual. Face to Face	Registered mental health professionals (psychological counselors and clinical psychologists) trained in TF-CBT	Abbreviated to 8 sessions to align with resource constraints in South African. Manual adapted to suit local context. The adapted manual emphasises asking participants to generate examples from their own life on which to practice the various skills, to ensure that practice examples are con-textually relevant for South African participants
McMullen et al. [67]	Group Trauma-focused CBT (25)	Waitlist (25)	A manualized Trauma Focused-CBT intervention focused on psychoeducation, relaxation techniques, affect expression, cognitive restructuring, trauma narration, and coping	15 sessions over five weeks	Group. Face to Face	Experienced foreign and local mental health experts	Manual modified to include culturally applicable analogies and exemplars. Local games and songs used to help the participants relax, learn social skills, and participate in group activities. Screening instrument modified to fit the context and language (Swahili) Use of local interpreters
O'Callaghan et al. [68]	Group Trauma-focused CBT (24)	Waitlist (28)	A manualized Trauma Focused-CBT intervention focusing on psychoeducation, stress management, affect expression and modulation, and cognitive restructuring and coping	15 sessions over five weeks	Group. Face to Face	Local (Congolese) social workers	Cultural adaptations included having a female facilitator talk about ways to reduce the risk of sexual violence (e.g., fetching firewood with a friend); the use of culturally familiar games, songs, and examples (e.g., belief that a neighbour is a witch); and social workers visiting the girl’s guardians to foster family acceptance and reduce stigmatization Screening Instrument modified to Swahili
Olashore et al. [71]	Psychoeducation and Problem-solving (25)	Waitlist (25)	The intervention involved interactive discussion, role-play, and brief plenary sessions. It included psychoeducation on HIV, depression, stigma, identifying problems, guided problem-solving, increasing pleasurable activities, mood monitoring, and rehearsing adaptive adherence strategies	1 h weekly sessions for 5 weeks	Group. Face to Face	Trained graduate psychology counsellor supervised by the principal investigator	Adapted from interventions designed in high-income countries using locally relevant video vignettes, illustrations and case examples in Setswana and English
Ofoegbu et al. [62]	Guided Internet Assisted Intervention (96)	Usual care (96)	A Guided internet-assisted intervention that covered psychoeducation, cognitive disputation, behavioural homework assignments, and role-play. The guidance therapists provided twice-a-week and on-request assistance within the portals, and through email and telephone. Guidance involved helping participants find useful resources	10 weeks	Individual. Internet-based	Guided self -help (Guidance by therapists)	None
Osborn et al. [65]	“Shamiri” Digital Intervention (50)	Study skills (53)	A Single Session Digital Intervention which consisted of three modules: growth mindset, gratitude, and value affirmation, based on principles of Wise Interventions. Study skills (control) consisted of two modules: note-taking skills and effective study habits	1 h Single session	Individual. Computer-based	Self-help	Local stakeholders (school administrators, students, and community members) were extensively involved in the development and adaptation process to ensure that the intervention was socio-culturally appropriate for the Kenyan context. Use of locally familiar examples and metaphors
Osborn et al. [64]	“Shamiri” Group Intervention (28)	Study skills (24)	A combination of three Wise Interventions (growth mindset, gratitude, and value affirmation). Each session included reading and writing activities, group discussions, and homework. Control group content focused on study skills such as note-taking, effective reading strategies, and time management	1 h weekly sessions for four weeks	Group. Face to Face	High school graduates trained as lay providers	Group discussions in either English or Kiswahili. Intervention adapted through an iterative process that took local culture and customs into consideration
Osborne et al. [66]	“Pre-Texts” Arts-literacy intervention (106)	Study skills. (129)	Creative Arts-literacy interventions employ art-based psychotherapeutic approaches to facilitate psychological change. Pre-Texts utilized text—such an “excerpt from a novel, a physics lesson, or a technical manual—to inspire art-making”. This is followed by a collective reflection on the process of interpretation through artmaking	1 h daily sessions for one week	Group. Face-to-Face	High school graduates trained as lay providers	Students were encouraged to use local arts, languages, traditions, and tastes as resources for learning to make the intervention more engaging and relevant to their own lives
Rivet-Duval et al. [73]	Resourceful Adolescent Program- Adolescent version (RAP-A) (80)	Waitlist (80)	A manualised universal based program which included both CBT and IPT approaches covering topics such as building self-esteem, keeping calm, thinking resourcefully, problem solving, considering the perspective of others and keeping the peace	1 h weekly sessions for 11 weeks	Group. Face to Face	School Teachers	None
Thurman et al. [69]	Group Interpersonal Psychotherapy (260)	Usual care (229)	Intervention utilized an IPT-G manual which focused on four interpersonal areas that trigger depressive symptoms: grief, interpersonal disputes, role transitions, and relationship deficits	1.5 h min weekly sessions for 16 weeks	Group. Face to Face	Lay facilitators recruited from the community	None
Tol et al. [72]	Group CBT techniques + Creative Expressive elements (153)	Waitlist (176)	A manualized intervention which consisted of cognitive behavioural techniques (psychoeducation, coping, trauma narration, future planning, social reconnection) and creative expressive elements (cooperative games, structured movement, music, drama, and dance)	15 sessions over five weeks	Group. Face to Face	Locally identified non-specialized facilitators	NR
Unterhitzenberger and Rosner [74]	Unstructured Emotional Writing (23)	Positive writing (23), No Writing (23)	Emotional Writing involved participants writing about their deepest emotions concerning their loss. Positive Writing participants were asked to write about a trivial but positive topic like their favourite hobbies	30 min weekly for three weeks	Individual. Face to Face	School Teachers	Adolescents received instructions and wrote in their mother tongue, Kinyarwanda

*NA* Not applicable, *NR* Not reported

### CBT-based interventions

All but two CBT-based studies tested manualized interventions delivered in group face-to-face format. One delivered CBT in an individual format [70] while the other was an online guided self-help intervention [62]. The two group Trauma-Focused CBT (TF-CBT) also included a few individual sessions for trauma narration to “prevent vicarious traumatization” [67, 68]. The different manuals used across studies included core CBT elements like psychoeducation, cognitive restructuring, activity scheduling, problem-solving, and relaxation techniques, as shown in Table 4. The manual used by Are et al. [55] was developed in Nigeria by one of the co-authors and used in two other Nigerian studies [56, 61]. This is in contrast with other studies which used manuals developed in Western countries. Two interventions added other elements to CBT. The Guided Internet Assisted Intervention (GIAI) combined CBT techniques with interactive peer support [62], while another study [72] added creative expressive elements to CBT.

In terms of intensity, all the interventions can be considered Low-intensity as they were either delivered in high volume (group format) and/or by non-mental health professionals, or as self-help. However, they varied in duration, with seven interventions lasting 5 weeks or less and six lasting 8 weeks or more (Table 4). Although some interventions lasted 5 weeks, they had multiple sessions per week (15 sessions overall) in contrast with others, which had one session per week (five sessions). Eight interventions were delivered by mental health professionals, while two were delivered by non-professionals (e.g., teachers and lay healthworkers). In the guided self-help, guidance was provided by therapists [62].

### Interpersonal psychotherapy

Like most CBT-based studies, the two IPT studies were manualized interventions delivered in group face-to-face format. One intervention was delivered to Ugandan adolescents displaced by war [75] while the other was for HIV orphaned adolescents in South Africa [69]. In both studies, 1.5-2 hr weekly sessions were delivered by lay facilitators for 16 weeks, using the same IPT manual developed by a humanitarian organization. Though one study [75] randomized participants to intervention (IPT), and two control groups (Creative Play and Waitlist), they only analyzed the IPT group against the waitlist group.

### Other intervention types: wise interventions and creative psychological interventions

Wise Interventions are a novel class of ordinary, briefer, and precise positive psychological interventions aimed at altering a specific way in which people think or feel [76]. The WIs (*Shamiri*) are the first positive psychology intervention to combine three WIs (Growth Mindset, Value Affirmation, and Gratitude). *Shamiri* means “thrive” in Kiswahili, which reflects the intervention’s focus on positive psychology rather than mental illness [64, 65]. The two WIs were conducted among low-income students in an urban slum in Kenya. One intervention [64] was delivered in a group format over 4 weeks by former high school graduates, while the other (Shamiri Digital) [65] was a single-session digital self-help intervention.

Three studies examined the effect of Creative Psychological Interventions on depressive symptoms. CPIs encompass a variety of psychotherapautic techniques that utilize creative and expressive forms of communication and expression to address psychological and emotional issues [77]. Two of these studies tested Expressive Writing. *Writing for Recovery* (WFR) [63] adopted a structured testimonial/narrative approach in communicating an emotional experience to normalize distressing reactions while *Emotional Writing* was an unstructured writing intervention for HIV-orphaned adolescents and involved participants writing about their deepest emotions concerning their loss [74]. The other CPI was conducted among high school students and employed art-based psychotherapeutic approaches to facilitate psychological change [66].

### Cultural/contextual adaptation

As shown in Table 4, sixteen studies reported some form of adaptation to suit the local culture and context, while six did not. The most common form of adaptation was the delivery of the intervention in the local language. This involved group discussions in the local language and/or translation of the screening instrument and manual. Three studies [67, 68, 75] used locally developed screening instruments and three used locally developed manuals [55, 56, 61]. Eseadi et al. adapted the intervention manual to incoporate the religious philosophies and traditions of the participants. Another common adaptation was the use of local metaphors, and culturally applicable analogies and exemplars. The two Trauma-Focused CBT in the DRC also used cultural games and songs [67, 68]. Are et al. encouraged the use of helpful cultural and religious coping mechanisms while Osborn et al. encouraged the use of local arts, languages and traditions [55, 66]. Three interventions ensured rigorous cultural appropriateness by involving community stakeholders in the development and implementation of the interventions [63–65].

### Risk of bias assessment

**Fig. 2:**
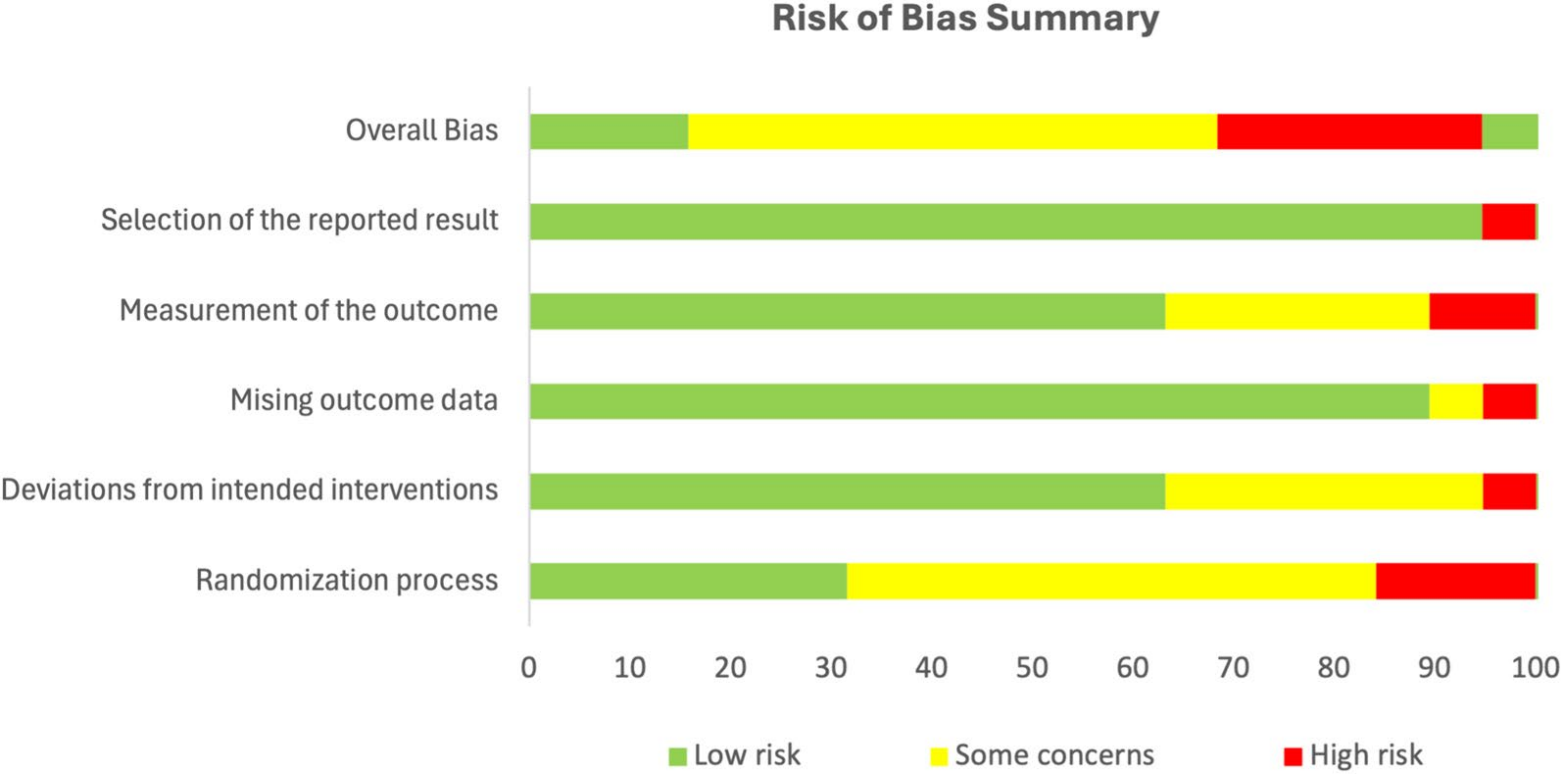
Risk of bias summary graph

**Fig. 3:**
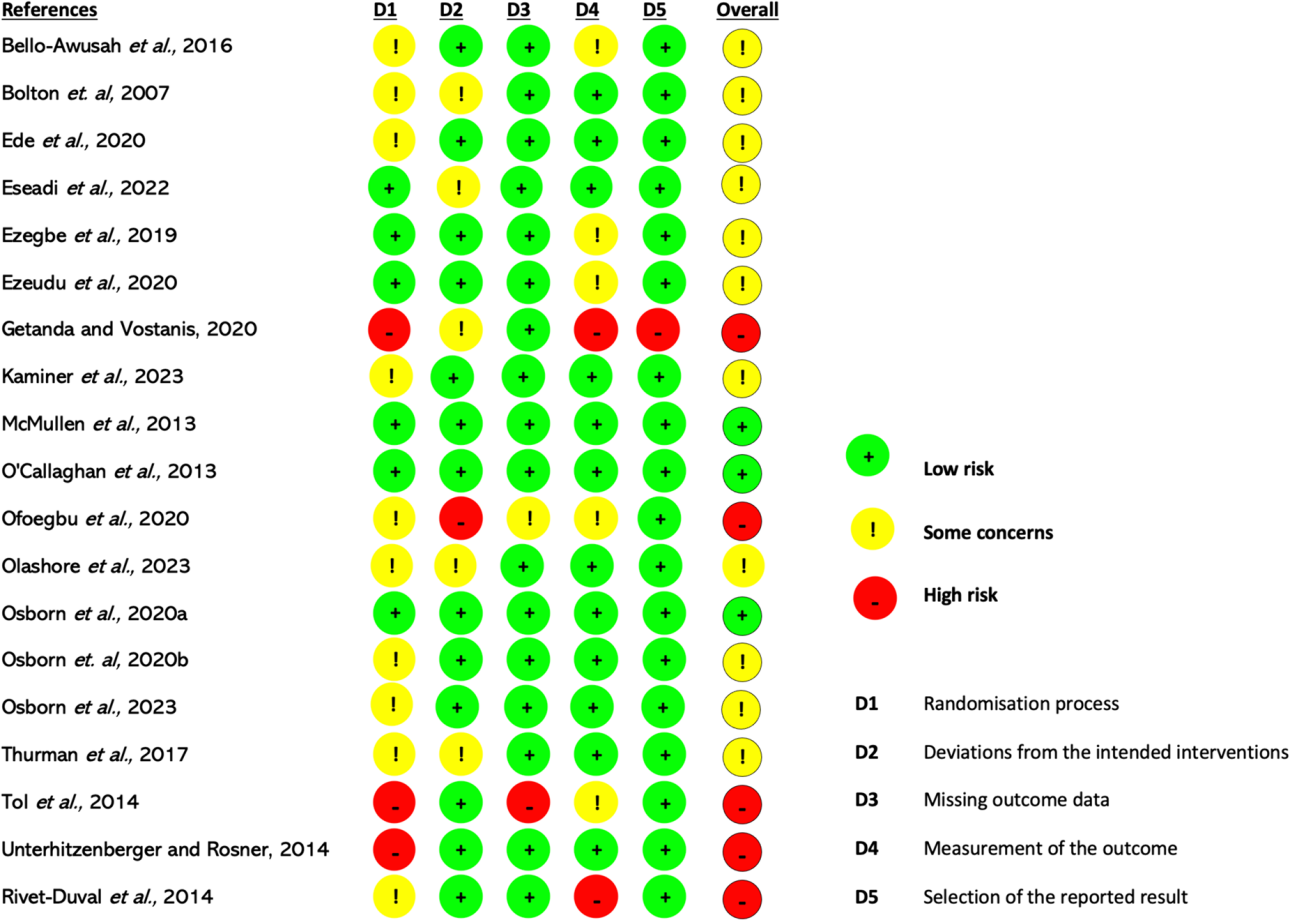
Risk of bias judgment for included RCTs

Eleven of the ninteen RCTs were judged as having “some concerns”. Five studies were judged high risk, while three had a low RoB (Fig. 3). Bias arose mainly from the ‘Randomization Process’ and ‘Measurement of the Outcome’ domains (Fig. 2).

Figure 3 presents the RoB for each RCT. In the Randomization Process, studies were judged high or had “some concerns” due to lack of information on allocation concealment [62, 64, 66, 69–73] and/or significant differences in prognostic variables between intervention and control groups [72, 74]. For instance, in one study, participants differed significantly in baseline depressive symptoms, social support, and experience of traumatic events [72]. In the Measurement of the Outcome domain, five studies [56, 59, 60, 62, 72] were judged to have “some concerns” due to unblinded outcome assessors. Two studies [63, 73] were rated high because unblinded intervention facilitators supervised the post-intervention completion of screening instruments, making outcome assessment more likely to be influenced by knowledge of allocation. For Deviation from Intended Intervention, one study [62] was rated high because it neither reported information on intervention fidelity nor whether participants were analyzed in the groups they were randomized to (e.g. using Intention-To-Treat(ITT) analysis). Furthermore, 41% of participants dropped out and were excluded from the outcome analysis. Five studies had “Some Concerns” due to lack of information on intervention fidelity and or analysis methods [58, 63, 73] or reported protocol deviations [69, 75]. In the Missing Outcome Data domain, most studies had low RoB. However, one study [67] was rated high due to potential outcome-related missing data. One study [61] had “some concerns” due to lack of missingness accounting and sensitivity analysis. Only one study [63] was judged as having a high RoB in the Selection of Reported Results Domain because it used multiple methods to assess treatment effects but reported only one set of results. For the Non-randomized studies, the two pre-post intervention studies [54, 61] had serious RoB while the controlled trial [55] had a low RoB (Table 5).

**Table 5:** Risk of Bias Judgement for Non-Randomized Studies of Interventions (NRSI)

References	Confounding	Selection of participants	Deviation from intervention	Missing data	Measurement of outcome	Selective reporting	Overall judgment	Reasons
Adegbolagun et al. [54]	Serious	Low	Low	Low	Moderate	Low	Serious	Possible confounding factors such as age and gender not adjusted for. Regression to the mean not accounted for. Self-reported measures used
Are et al. [55]	Low	Low	Low	Low	Low	Low	Low	Use of control group largely eliminated confounding bias
Isa et al. [61]	Serious	Low	Low	Low	Moderate	Low	Serious	Possible confounding factors like age, gender, education, SES, not measured and controlled for. Also does not use appropriate statistical methods to account for regression to the mean. Self-reported outcome could lead to response bias

### Effect of psychosocial interventions

Table 6 shows the results of each study, including baseline and post-intervention mean depression scores, treatment effect and factors affecting efficacy. Overall, 19 studies, of which 12 were CBT-based, reported statistically significant reductions in depressive symptoms in the intervention compared to control groups. Although the follow-up durations varied between studies, majority of the CBT interventions (nine) maintained their effects at follow-up. Although both IPT interventions were similar (same manual, duration, and delivery personnel), results were mixed. Bolton et al. [75] reported a significant decrease from baseline depressive symptoms (P = 0.02) only amongst girls, while Thurman et al. [69] revealed no significant decrease (P = 0.145). Despite the differences in their delivery formats, both Wise Interventions reported significant decreases from baseline mean depression scores in intervention groups compared to control [64, 65]. Interestingly, the single-session digital version (Shamiri Digital) [65] showed a more significant reduction and larger effect size (P = 0.028, Cohen’s d = 0.5) than the four-session group face-to-face version (Shamiri Group)(64) (P = 0.038, d = 0.32). Two of the three creative psychological interventions showed significant effects. “Pre-Texts”, the arts-based therapy showed a significant reduction in depression scores (P = 0.001, d = 0.52). The structured writing intervention, Writing for Recovery (WFR) [63] resulted in a significant decrease in depressive symptoms (P = 0.0001) with a large effect size (np^2^ = 0.338). In contrast, the unstructured writing intervention showed no significant change from baseline (P = 0.518) [74]. The two other interventions had small effect sizes. The universal preventive intervention, Resourceful Adolescent Programme (RAP) [73], resulted in a significant decrease from baseline depressive symptoms (P < 0.001), with a small effect size (d = 0.32). However, the effects were not maintained at 6-months follow-up. The intervention which combined psychoeducation and problem-solving also yielded a small effect (ƞp^2^ = 0.20, p = 0.001). Overall, both culturally adapted and non-adapted interventions showed positive effects.

**Table 6:** Results

References	Intervention group mean depression scores (Standard Deviation)		Control group mean depression scores (Standard Deviation)		Treatment effect (P value)	Effect size	Factors affecting efficacy
	Baseline	Post-intervention	Baseline	Post-Intervention			
Adegbolagun et al. [54]	4.50 (2.81)	2.83 (1.92)	NA	NA	P < 0.02	NA	Increase in coping as a possible mediator
Are et al. [55]	24.30 (6.59)	4.60 (7.35)	24.25 (6.06)	17.05 (11.17)	P = 0.0001	np^2^ = 0.32	Age, gender, religion, and parent’s level of education did not predict outcome. Increased self esteem and knowledge of depression as possible mediators
Bella-Awusah et al. [56]	25.3 (8.8)	11.8 (9.5)	24.2 (6.1)	21.1 (7.9)	P = 0.0001	ƞp^2^ = 0.31	NR
Bolton et al. [75]	43.5 (10.1)	27.8 (17.2)	44.2 (10.8)	37.3 (15.8)	P = 0.02	NR	Sex predicted the outcome. Age did not predict outcome. Increased adherence was associated with reduction in symptoms
Ede et al. [57]	49.10 (6.25)	12.45 (6.61)	50.44 (7.68)	49.30 (4.99)	P < 0.001	ƞp^2^ = 0.88	NR
Eseadi et al. [58]	40.15 (4.24)	12.91 (5.70)	41.97 (4.92)	44.39 (5.56)	P < 0.0001	ƞp^2^ = 0.82	NR
Ezegbe et al. [59]	68.57 (10.06)	14.68 (1.63)	65.11 (13.06)	64.63 (17.13)	P = 0.0001	ƞp^2^ = 0.91	Level of adherence did not moderate treatment outcome
Ezeudu et al. [60]	46 (6)	24 (4)	43 (5)	42(4)	P < 0.001	ƞp^2^ = 0.916	NR
Getanda and Vostanis [63]	18.1 (6.7)	10 (6.9)	15.8 (5.3)	17.0 (5.5)	P = 0.0001	np^2^ = 0.338	NR
Isa et al. [61]	24.4 (11.18)	3.94 (2.10)	NA	NA	P = 0.001	NA	Increased Hope and knowledge of depression as a possible mediator
Kaminer et al. [70]	26.97 (14.96)	15.44 (14.16)	26.79 (13.27)	23.44 (17)	P = 0.03	d = 0.51	NR
McMullen et al. [67]	38.1 (9.3)	7.0 (5.8)	38.1 (11.1)	29.3 (13.6)	P < 0.001	ƞp^2^ = 0.567	NR
O'Callaghan et al. [68]	37.96 (10.16)	13.96 (10.30)	39.18 (10.57)	40.04 (15.18)	P < 0.001	ƞp^2^ = 0.517	NR
Ofoegbu et al. [62]	65.30 (2.61)	8.60 (0.21)	64.78 (3.67)	65.50 (2.4)	P = 0.0001	ƞp^2^ = 0.959	NR
Olashore et al. [71]	13 (2.0)	7 (3.0)	13 (3.0)	9 (2.0)	P = 0.001	ƞp^2^ = 0.20	NR
Osborn et al. [65]	14.43 (3.35)	8.46 (4.98)	13.18 (2.89)	10.64 (4.89)	P = 0.028	d = 0.5	Younger adolescents reported larger declines in depressive symptoms. Adolescents with moderate to severe depression had larger symptom reduction
Osborn et al. [64]	13.43 (4.14)	10.21 (4.39)	12.91 (3.52)	12.52 (4.23)	P = 0.038	d = 0.32	Age and sex were not associated with outcome
Osborn et al. [66]	8.29 (5.30)	5.69 (4.23)	8.40 (4.69)	8.37 (5.53)	P = 0.001	d = 0.52	NR
Rivet-Duval et al. [73]	51.8 (9.07)	47.5 (7.95)	50.61 (9.19)	49.98 (11.07)	P < 0.001	d = 0.32	NR
Thurman et al. [69]	16.03 (1.39)	16.46 (1.92)	14.67(1.26)	15.63 (2.12)	P = 0.145	NE	Age and sex showed no moderation effect on results
Tol et al. [72]	9.97 (4.82)	8.15(5.02)	11.28 (5.08)	8.49 (5.8)	P = 0.149	NE	Larger households and having both parents were effect moderators
Unterhitzenberger and Rosner [74]	18.8 (10.60)	20.6 (11.4)	15.7 (9.9)	12.1 (10.3)	P = 0.518	NE	NR

*NA* Not applicable, *NR* Not reported, *NE* No effect

Measures of effect: *np*^2^, Partial Eta Squared, *d* Cohen’s D

### Meta-analysis

**Fig. 4:**
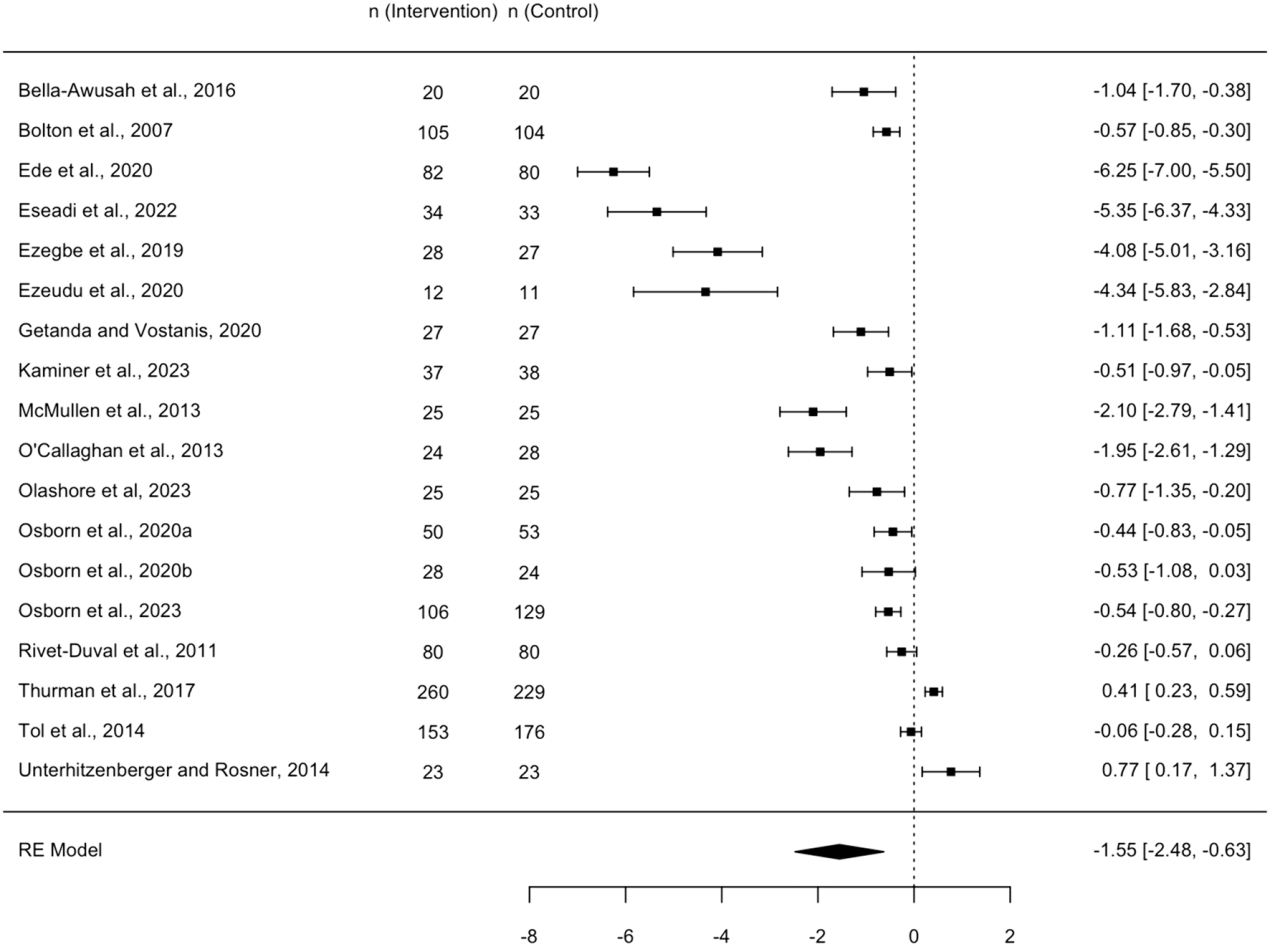
Forest plot – effects of Psychosocial Interventions of depressive symptoms. Negative effect sizes signify a more favorable outcome (symptom reduction) for the psychosocial intervention groups relative to the control groups

The meta-analysis incorporated data from 18 RCTs involving 2338 participants. One study, identified as an extreme outlier due its exceptionally large effect size (Hedges’ g = −33) and high risk of bias was excluded from the analysis [62]. The random effects model revealed a significantly large effect of psychosocial interventions compared to the control groups (Hedges’ g = −1.55, 95% CI −2.48, −0.63). Figure 4 presents the forest plot, displaying the effect sizes (Hedges’ g) for each study, the pooled effect size and 95% confidence intervals. Negative effect sizes indicate a more favorable outcome (symptom reduction) for the psychosocial intervention groups relative to the control groups. The analysis revealed high heterogeneity among the included studies (I^2^ = 98.8%, Q = 574, p < 0.0001), indicating significant variability. Sensitivity analyses, excluding studies with a high risk of bias amplified the effect size (Hedges’ g = −1.96, 95% CI −3.5 to −0.86), although the heterogeneity remained unchanged. However, removing the four outliers whose effect sizes were much larger than the others (g > 4) resulted in a reducted effect size (g = −0.59, 95% CI −0.98, −0.2) and decreased heterogeneity (I^2^ = 93.8%). Interestingly, these outliers were the group CBT interventions with the longest duration (12 weeks) and were all conducted among undergraduate students in Nigeria [57–60]

**Fig. 5:**
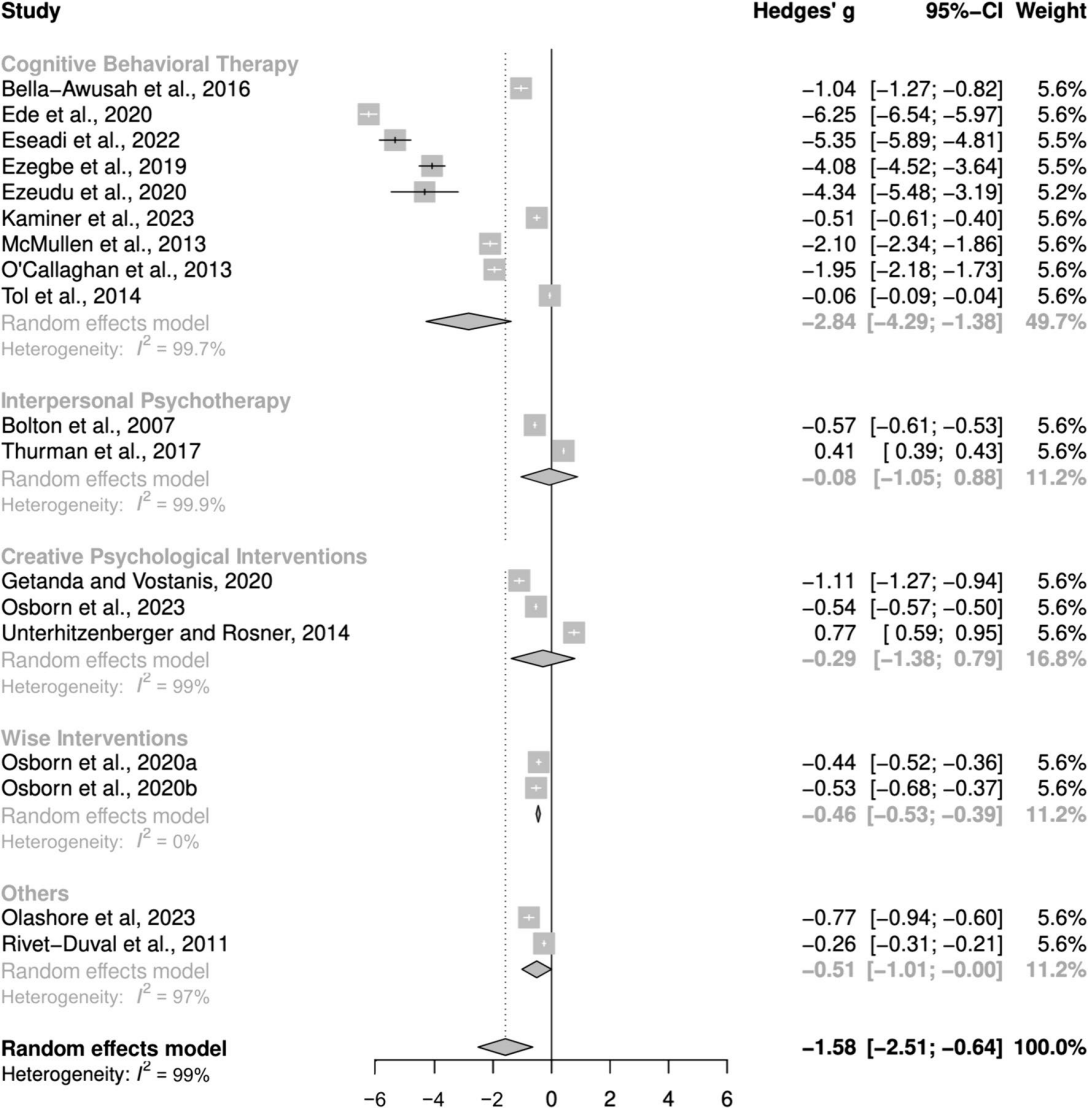
Forest plot for sub-group analysis

Subgroup analysis, depicted in Fig. 5, indicated significant differences in effect size by intervention type (Test for subgroup differences: p = 0.027). CBT-based interventions represented in nine studies, had the strongest effect (Hedges’ g = −2.84, 95% CI −4.29; −1.38), albeit with high within-group heterogeneity (I^2^ = 99.7%). Other subgroups, with fewer studies, exhibited smaller average effects. Wise Interventions had a moderate effect (g = −0.46, 95% CI −0.53, −0.39) while Interpersonal Psychotherapy (g = −0.08, 95% CI −1.05, 0.88) and Creative Psychological Interventions (g = −0.29, 95% CI −1.38, 0.79) showed small non-significant effects. Within-group heterogeneity was high for IPT and CPI (99.9% and 99%, respectively), while WI showed no heterogeneity (I^2^ = 0%).

**Fig. 6:**
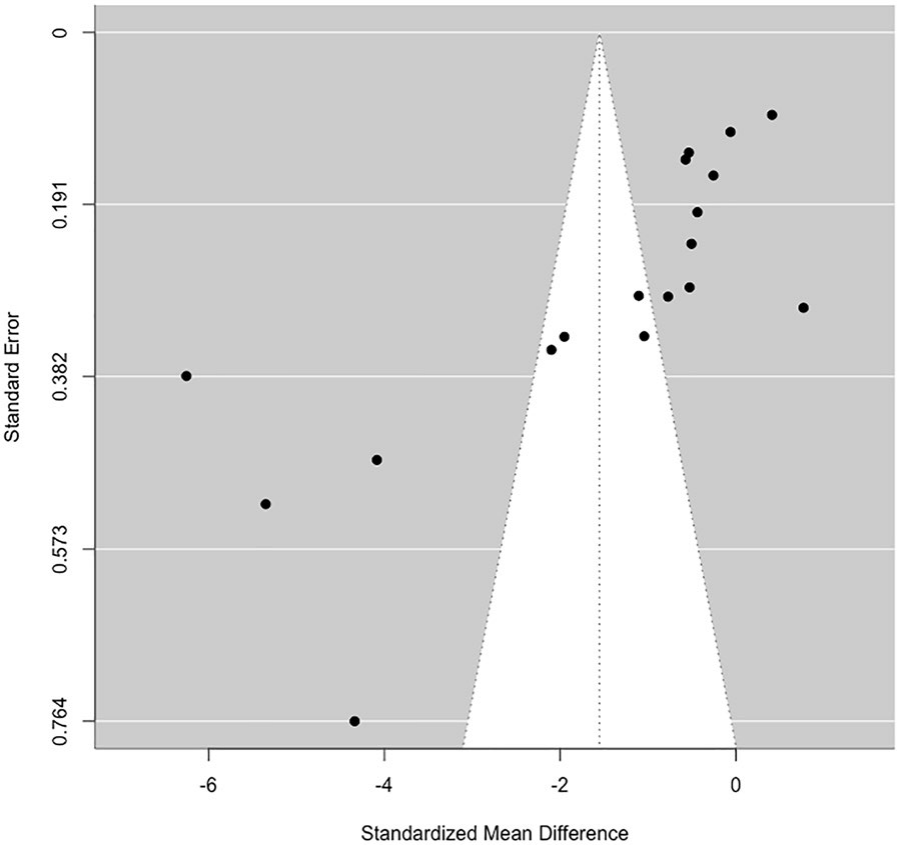
Funnel plot for assessing publication bias. The observed funnel plot asymmetry should be interpreted cautiously, considering the high heterogeneity among the included studies

Publication bias was assessed using funnel plots and Egger’s test, which indicated significant funnel plot asymmetry (p < 0.0001), raising concerns about potential publication or small study bias (Fig. 6). However, Duval & Tweedie’s trim and fill analysis estimated no missing studies. The pooled effect size was maintained after the small-study bias adjustment, suggesting the robustness of the findings [78].

### Factors affecting intervention efficacy: predictors, moderators and mediators

As shown in Table 5, majority of studies neither assessed potential predictors nor conducted moderator and mediation analysis. Three studies conducted statistical analysis to determine what baseline variables would predict outcomes [55, 64, 75]. Gender predicted the outcome in one study [75], as the intervention was only effective for girls, but it was not an outcome predictor in the others [55, 64]. Age did not predict outcome in any of the three studies.

Age moderated the efficacy of Shamiri Digital [65], as younger adolescents reported a larger decline in depressive symptoms. Contrastingly, neither age nor gender were moderators in the study by Thurman et al. [69]. Results for level of adherence as a moderator were also mixed, as increased adherence was associated with symptom reduction in one study [75] but not in another [59]. The severity of depression was an effect moderator in Shamiri Digital as there was better response among adolescents with moderate-to-severe depression scores [65].

Increase in coping, self-esteem, hope, and knowledge of depression were identified as possible mediators [54, 55, 61].

## Discussion

This systematic review is the first to assess the efficacy of psychosocial interventions for depression among young people in Sub-Saharan Africa (SSA). The meta-analysis showed that these interventions, particularly CBT, significantly reduce depressive symptoms, although substantial heterogeneity necessitates cautious interpretation of pooled estimates. Subgroup analysis indicated significant variation in efficacy by intervention type. CBT was shown to be the most effective intervention, corroborating findings from systematic reviews in other contexts [21, 38]. Wise Interventions (WI) showed moderate effects, while IPT and CPI had small effects. However, limited number of studies per group may affect the reliability of the subgroup estimates. The WIs particularly the single session intervention (SSI) had effect sizes comparable to some well-established psychological treatments, which is interesting because WIs were not originally developed for depression [65, 76]. A similar study in the US found a digital single-session WI to be moderately effective in reducing adolescent depressive symptoms [79]. As positive psychology interventions, WIs could help address stigma, which is a major barrier to treatment in SSA. However, more studies are needed to ascertain the durability and reproducibility of their effects. A review by Weersing et al. [38] found IPT to be effective among young people but less so in group format, which could explain the lower efficacy of Group-IPT compared to Group CBT in our review. Furthermore, the effective Group-IPT studies from their review were conducted among the general population, as opposed to war-affected adolescents in this review. In contrast, Group TF-CBT studies showed large effects, suggesting that they might be better suited than IPT for trauma-affected adolescents in SSA.

### Exploring sources of heterogeneity

While the sensitivity analysis and publication bias adjustment suggest robust findings, the high overall and subgroup heterogeneity necessitate cautious interpretation of pooled effect sizes. Thus, it might be more informative to focus on understanding the sources of variation across studies rather than relying solely on the pooled estimate. Subgroup analysis revealed high within-group heterogeneity for all subgroups except WI, indicating that variability extends beyond intervention types. Differences in study population and design, and intervention delivery may contribute to this variability. For instance, within CBT studies, heterogeneity likely arises from differences in participant characteristics, and intervention delivery methods. Studies with homogenous characteristics and delivery modalities, such as the culturally adapted group trauma-focused CBT (TF-CBT) for war-affected adolescents in DRC [67, 68], showed consistent effect sizes (g = −2.10, −1.95). In contrast, the individual TF-CBT for South African adolescents with moderate and severe PTSD had a smaller effect size (g = −0.51). The comparatively smaller effect size is attributable to the study’s inclusion of more clinically severe cases and its use of a treatment-as-usual control compared to the non-clinical population and waitlist control in the DRC studies. The group CBT intervention delivered to war-affected secondary school students in Burundi exhibited a much lower effect size (g = −0.06) than the other studies involving trauma-affected youths, likely due to its non-trauma-focused approach [72].

The other CBT interventions targeted general populations of high school and undergraduate students and also exhibited varying effects likely due to dissimilar population, inclusion criteria and intervention duration. For example, the extensive group CBT studies (12 week duration) delivered to a general population of undergraduate students in Southeast Nigeria [57–60] yielded much larger effect sizes (g =−4.08 to −6.25) compared to the shorter interventions (5 weeks) delivered to clinical populations of secondary school students in Southwest Nigeria (g = −1.04, −1.26) [55, 56].

The two group IPT trials showed disparate effects potentially due to differences in cultural adaptation and trauma exposure. Culturally adapted IPT for war-displaced adolescents had a moderate effect size [75] while non-adapted IPT delivered to HIV-orphaned adolescents showed no effect [69]. The CPI subgroup also had significant heterogeneity, possibly stemming from variations in participant and intervention characteristics. For instance, the structured EW intervention was effective for displaced secondary school students in Kenya [63], while unstructured EW showed no effect for orphaned adolescents in Rwanda [74]. Further studies are needed to ascertain the efficacy of EW as an intervention for depression and whether structured EW is more effective than unstructured. Unlike other subgroups, WIs showed no heterogeneity, likely because both studies were conducted by the same authors with similar participants and settings.

### Durability of effect and factors affecting efficacy

The intervention effects were generally maintained at follow-up, but many studies had short or no follow-up periods. Considering the high relapse and recurrence rates among adolescents [83], future research is needed to determine the durability of effects [80]. Additional studies are also required to uncover factors influencing intervention efficacy. While age, gender and adherence level were assessed in few studies, their predictive or moderating roles varied. A systematic review had similarly mixed findings on predictors and moderators of efficacy [38]. This gap is a key area of focus for future research as identifying these factors can lead to the development of tailored interventions. The role adaptation plays in efficacy is also worth exploring. Generally, evidence from this review is mixed as with other reviews. In their review, Cuijpers et al*.* did not find any indication that a specific contextual adaptation was associated with better outcomes [47]. Contrarily, another systematic review found culturally adapted interventions more effective than non-adapted ones but it was unclear what specific adaptations were important [81]. Since cultural adaptation can increase acceptability and adherence which are both associated with increased effectiveness [82], future studies should examine what adaptations are beneficial in different SSA countries.

### Intervention delivery modalities

All the interventions can be classified as Low-Intensity Psychological Interventions (LIPIs) due to reduced usage of therapist’s time (fewer sessions and/or high-volume delivery in group format), delivery by non-professionals, or as digital self-help interventions [83]. Compared to HICs and LMICs, this review found more interventions delivered in group format [21, 38]. Though group interventions are resource-effective hence better-suited for SSA, they are not suitable for everyone. For example, people with social phobia, interpersonal problems, recent traumatic events, and actively suicidal patients might be better served by individual therapy [84]. More studies are required to determine which people are better served by group vs individual interventions. Innovative approaches like embedding individual sessions in group interventions as done in the TFT-CBT studies [67, 68] can be further explored as they could prove more cost-effective. Interventions delivered by both mental health professionals and non-professionals were found to be effective. This concurs with a systematic review which found psychosocial interventions delivered by lay facilitators to be effective [85]. This finding is important for mental health policy in SSA as the region has the lowest ratio of mental health workers per population in the world [22]. Other novel innovative LIPIs were digital self-help and single session interventions (SSIs). Both self-help Digital Mental Health Interventions (DMHI) proved effective despite different durations (10-weeks vs single-session) and approaches (CBT-based vs WI/positive psychology). The evidence base for DMHI is growing and reviews in other contexts have found DMHI like computerized CBT to be effective in reducing adolescent depression [86]. An added advantage of digital SSIs is their ability to circumvent the high attrition often seen in multisession interventions [65]. Digital self-help interventions represent an opportunity to increase treatment access to young people in SSA due to increasing access to digital technology [87]. DMHI are also resource-effective and can help combat stigma thus warrant further exploration in the region.

### Limitations

The high heterogeneity observed in this study introduces challenges to the certainty and generalizability of the pooled effect size estimate. This variability among included studies suggests that combining their effect sizes may not be ideal, as they might measure different effect sizes based on study population. However, interventions between studies varied substantially (e.g., content, frequency, duration, adaptation and delivery personnel), suggesting that high heterogeneity may be inevitable.

Publication bias remains possible even though no missing studies were estimated. Notably, the lack of change in effect size after adjustment raises the possibility that funnel plot asymmetry may be attributable to between-study heterogeneity rather than small-study bias. Funnel plots, by assuming that the dispersion of effect sizes is due to sampling error, do not control for the fact that studies may be estimators of different true effects, further underscoring the importance of interpreting the pooled effect size in light of heterogeneity [50].

Another important limitation is the inability to assess the durability of effects due to inconsistent reporting of follow-up time across included studies.

## Conclusion

This study provides evidence supporting the efficacy of psychosocial interventions, particularly CBT, in alleviating depressive symptoms among young people in SSA. However, the observed heterogeneity highlights the importance of considering intervention types, delivery modalities, participant populations and factors affecting efficacy. Thus, while psychosocial interventions show promise in reducing youth depressive symptoms in SSA populations, further research to identify components that work best for specific subgroups is imperative. Tailored interventions for specific populations may be more effective than a one-size-fits-all approach.

### Supplementary Information

Supplementary material 1. Prisma checklist.

Supplementary material 2. Systematic review protocol.

Supplementary material 3. Search strategy.

Supplementary material 4. Reasons for exclusion.

## Data Availability

Data sharing is not applicable to this article as no datasets were generated or analysed during the current study.

## Abbreviations

APAI: Acholi psychosocial assessment instrument
AYPAI: African youth psychosocial assessment instrument
BDI: Beck’s depression inventory
CBT: Cognitive behavioural therapy
CMD: Common mental disorders
CCT: Controlled clinical trial
CPI: Creative psychological interventions
DMHI(s): Digital Mental Health Intervention(s)
DRC: Democratic Republic of Congo
EW: Expressive writing
g: Hedge’s g
GBD: Global burden of disease
GIAI: Guided internet assisted intervention
HICs: High Income Countries
IPT: Interpersonal psychotherapy/psychotherapy
ITT: Intention to treat
LMICs: Low- and Middle-Income Countries
LIPI: Low intensity psychological interventions
ƞp^2^: Partial Eta squared
NRSI: Non-randomized studies of interventions
PICOS: Population, intervention, comparison, outcome, study design
PTSD: Post-traumatic stress disorder
RAP: Resourceful adolescent programme
RCT: Randomized control trial
RoB: Risk of bias
SA: South Africa(n)
SSA: Sub-Saharan Africa
SSI: Single session intervention
TF-CBT: Trauma-focused cognitive behavioural therapy
WFR: Writing for recovery
WHO: World Health Organization
WI: Wise intervention
