# Supplementary material for: Psychosocial interventions for depression among young people in Sub-Saharan Africa: a systematic review and meta-analysis
Source: Int J Ment Health Syst. 2024 Jun 22;18:24. doi: 10.1186/s13033-024-00642-w (PMC11193191; doi:10.1186/s13033-024-00642-w)
Supplement: Supplementary file 2 — Supplementary material 2. Systematic review protocol. [file 13033_2024_642_MOESM2_ESM.docx]

**Additional File 2: Systematic Review Protocol**

| **Title of Review** | A Systematic Review of Psychosocial Interventions for Depression among Young People in Sub-Saharan Africa (SSA) |
| --- | --- |
| **Background** | |
| Depression affects 280 million people globally and is the second leading cause of disability worldwide^1,2^. The global prevalence of depression has been rising in previous years, with the COVID-19 pandemic resulting in a 28% increase^3^. Depression is the second most prevalent mental disorder among young people^2^ (people between ages 15 and 24)^4^. Adolescent and youth depression necessitates increased attention due to the first appearance of depression around this period^5^, its rising prevalence^6–8^, and associated negative physical and social outcomes such as suicide, substance use, risky sexual behavior, youth delinquency, and poor educational attainment^9,10^. Despite these, about eighty percent of young people with common mental disorders in LMICs do not get the care they need^11^.  Sub-Saharan Africa (SSA) consists of the forty-eight African countries that lie south of the Sahara. It is the poorest region in the world and contains twenty-four of the twenty-seven countries in the World Bank’s Low-Income Country classification^12^. SSA has the youngest population in the world with a third of its population under 30^13^. The estimated prevalence of adolescent and youth depression in most SSA countries is higher than the global average^14,15^. This is possibly due to regionally prevalent risk factors like poverty, poor education, HIV infection, sexual violence, and teenage pregnancy. Yet, most young people with common mental disorders in SSA do not receive the care they need and only nine of the forty-eight countries have policies for adolescent mental health^16^. This prompted the World Health Organization in its landmark report to call for the development and implementation of interventions for adolescent mental health in SSA^17^.  Psychosocial Interventions (PIs) are the first-line approach to depression in young people^18^. PIs like Cognitive Behavioural Therapy and Interpersonal Therapy have been shown to be effective for adolescent depression^19–21^. However, most of the studies that established the efficacy of these interventions were conducted in High-Income Countries. Interventions developed in High-income countries might not be acceptable, feasible, or effective in LMICs due to dissimilar cultural perceptions of depression, and barriers to care such as insufficient mental health workers, stigma, and poverty^22,23^. Systematic reviews of LMICs include only a few SSA countries, limiting their applicability to the region. To my knowledge, no systematic review has been conducted on the effectiveness of these PIs for depression among young people in SSA. | |
| **Review Question** | What psychosocial interventions are effective in reducing depressive symptoms among young people in Sub-Saharan Africa? |
| **Specific objectives** | 1. To identify and describe psychosocial interventions that are effective in reducing depressive symptoms among young people in Sub-Saharan Africa. 2. To explore factors affecting the effectiveness of these interventions. 3. To suggest directions for future research and policy. |
| **Inclusion criteria** | Experimental and Quasi-experimental studies that report the effect of various PIs on depressive symptoms in any adolescent or youth population (persons aged 15-24 years) in SSA. |
| **Exclusion criteria** | Papers that focus on non SSA countries and on people outside the 15-24 age group; grey literature, non-academic papers (commentaries, editorials, etc.), and books. |
| **Search methods** | |
| **Electronic databases** | PubMed/Medline  Web of Science  PsychInfo  Cochrane Central Register of Randomized Control Trials (CENTRAL). |
| **Other methods used to identify relevant research** | Snowballing method (searching references of included papers). |
| **Search terms** | |
| **Population**  **Intervention**  **Outcome** | **Keyword: Young People**  MeSH: **Adolescent**  Synonyms: Adolescent* OR Teenager* OR Teen* OR youth* OR young person OR young people OR youngster* OR young adult* OR student* OR high school OR college OR secondary school  **Keyword: Sub-Saharan Africa**  MeSH: **Africa South of the Sahara**  Synonyms: Sub-Saharan Africa OR SSA OR West* Africa OR East* Africa OR South* Africa OR Central Africa OR (*list of all SSA countries*).  **Keyword: Psychosocial**  MeSH: **Psychotherapy**  Synonyms: psychosocial OR psychological OR psychoeducation OR behavioural therapy OR cognitive behavioural therapy OR CBT OR Interpersonal Psychotherapy OR IPT OR problem-solving therapy OR counselling OR narrative therapy  **Keyword: Intervention**  Synonyms: Treatment OR Therapy OR Trial OR Project OR Program*  **Keyword: Depression**  MeSH: **Depression; Depressive Disorder**  Synonyms: depression or depressive disorder* or dysthymia or depressive symptom |
| **Methods of review** | |
| **Details of methods** | The keywords with their MeSH terms and synonyms will be combined with Boolean operators [“AND” and “OR”] and wildcards to run a comprehensive search on Medline (OVID). This search strategy will then be adapted to Web of Science, PsychInfo and Cochrane Central Register of Controlled Trials (CENTRAL). EndNote 20 will be used for de-duplication and to aid the screening process. |
| **Risk of Bias Analysis** | The Cochrane Risk of Bias tools for randomized control trials (RCTs) version 2 (RoB2) and Risk of Bias in Non-randomized Studies of Interventions (ROBINS-I) will be used to assess the risk of bias (RoB) for RCTs and Non-Randomized Studies of Interventions (NRSI)/Quasi-experimental studies respectively. |
| **Data extraction** | A data extraction form will be developed to extract relevant information from the papers such as country, study design, setting, screening instrument, intervention characteristics, and outcome (depressive symptoms pre- and post-intervention). |
| **Narrative synthesis** | The review will be a narrative synthesis which will:   1. Present the search results and describe the included studies. 2. Provide detailed reasons for Risk of Bias Judgment 3. Describe the important characteristics of studied interventions. 4. Describe any cultural and contextual adaptations of the interventions. 5. Present results on the effectiveness of the interventions in the included studies. |

**References**

1.  *Depression.* World Health Organization. Accessed July 4, 2022. https://www.who.int/news-room/fact-sheets/detail/depression

2. Global, regional, and national burden of 12 mental disorders in 204 countries and territories, 1990–2019: a systematic analysis for the Global Burden of Disease Study 2019. *The Lancet Psychiatry*. 2022;9(2):137-150. doi:10.1016/S2215-0366(21)00395-3

3. Santomauro DF, Herrera AMM, Shadid J, et al. Global prevalence and burden of depressive and anxiety disorders in 204 countries and territories in 2020 due to the COVID-19 pandemic. *The Lancet*. 2021;398(10312):1700-1712. doi:10.1016/S0140-6736(21)02143-7

4.  *Definition of Youth*. United Nations. Accessed July 13, 2022. https://www.un.org/esa/socdev/documents/youth/fact-sheets/youth-definition.pdf

5. Beirão D, Monte H, Amaral M, Longras A, Matos C, Villas-Boas F. Depression in adolescence: a review. *Middle East Current Psychiatry*. 2020;27(1):50. doi:10.1186/s43045-020-00050-z

6. Racine N, McArthur BA, Cooke JE, Eirich R, Zhu J, Madigan S. Global Prevalence of Depressive and Anxiety Symptoms in Children and Adolescents During COVID-19: A Meta-analysis. *JAMA Pediatrics*. 2021;175(11):1142-1150. doi:10.1001/jamapediatrics.2021.2482

7. Patalay P, Fitzsimons E. Development and predictors of mental ill-health and wellbeing from childhood to adolescence. *Soc Psychiatry Psychiatr Epidemiol*. 2018;53(12):1311-1323. doi:10.1007/s00127-018-1604-0

8. Patalay P, Gage SH. Changes in millennial adolescent mental health and health-related behaviours over 10 years: a population cohort comparison study. *International Journal of Epidemiology*. 2019;48(5):1650-1664. doi:10.1093/ije/dyz006

9. Pozuelo JR, Desborough L, Stein A, Cipriani A. Systematic Review and Meta-analysis: Depressive Symptoms and Risky Behaviors Among Adolescents in Low- and Middle-Income Countries. *Journal of the American Academy of Child & Adolescent Psychiatry*. 2022;61(2):255-276. doi:10.1016/j.jaac.2021.05.005

10. Clayborne ZM, Varin M, Colman I. Systematic Review and Meta-Analysis: Adolescent Depression and Long-Term Psychosocial Outcomes. *Journal of the American Academy of Child & Adolescent Psychiatry*. 2019;58(1):72-79. doi:10.1016/j.jaac.2018.07.896

11. Stelmach R, Kocher EL, Kataria I, Jackson-Morris AM, Saxena S, Nugent R. The global return on investment from preventing and treating adolescent mental disorders and suicide: a modelling study. *BMJ Global Health*. 2022;7(6):e007759. doi:10.1136/bmjgh-2021-007759

12. World Bank Country and Lending Groups – World Bank Data Help Desk. Accessed July 18, 2022. https://datahelpdesk.worldbank.org/knowledgebase/articles/906519-world-bank-country-and-lending-groups

13.  Youth Development. African Union. Accessed July 16, 2022. https://au.int/en/youth-development

14. Jörns-Presentati A, Napp AK, Dessauvagie AS, et al. The prevalence of mental health problems in sub-Saharan adolescents: A systematic review. *PLOS ONE*. 2021;16(5):e0251689. doi:10.1371/journal.pone.0251689

15. Cortina MA, Sodha A, Fazel M, Ramchandani PG. Prevalence of Child Mental Health Problems in Sub-Saharan Africa: A Systematic Review. *Archives of Pediatrics & Adolescent Medicine*. 2012;166(3):276-281. doi:10.1001/archpediatrics.2011.592

16. Mental Health ATLAS 2017. World Health Organization. Accessed July 16, 2022. https://www.who.int/publications-detail-redirect/9789241514019

17. World mental health report: Transforming mental health for all. World Health Organization. Published 2022. Accessed July 14, 2022. https://www.who.int/publications-detail-redirect/9789240049338

18.  Position statement on antidepressants and depression. Royal College of Psychiatry. Accessed July 16, 2022. https://www.rcpsych.ac.uk/docs/default-source/improving-care/better-mh-policy/position-statements/ps04_19---antidepressants-and-depression.pdf?sfvrsn=ddea9473_5

19. Kaslow NJ, Thompson MP. Applying the criteria for empirically supported treatments to studies of psychosocial interventions for child and adolescent depression. *Journal of Clinical Child Psychology*. 1998;27(2):146-155. doi:10.1207/s15374424jccp2702_2

20. David-Ferdon C, Kaslow NJ. Evidence-based psychosocial treatments for child and adolescent depression. *J Clin Child Adolesc Psychol*. 2008;37(1):62-104. doi:10.1080/15374410701817865

21. Weersing VR, Jeffreys M, Do MCT, Schwartz KTG, Bolano C. Evidence Base Update of Psychosocial Treatments for Child and Adolescent Depression. *Journal of Clinical Child & Adolescent Psychology*. 2017;46(1):11-43. doi:10.1080/15374416.2016.1220310

22. Uppendahl JR, Alozkan-Sever C, Cuijpers P, de Vries R, Sijbrandij M. Psychological and Psychosocial Interventions for PTSD, Depression and Anxiety Among Children and Adolescents in Low- and Middle-Income Countries: A Meta-Analysis. *Frontiers in Psychiatry*. 2020;10. Accessed July 17, 2022. https://www.frontiersin.org/articles/10.3389/fpsyt.2019.00933

23. Mabunda D, Oliveira D, Sidat M, et al. Cultural adaptation of psychological interventions for people with mental disorders delivered by lay health workers in Africa: scoping review and expert consultation. *International Journal of Mental Health Systems*. 2022;16(1):14. doi:10.1186/s13033-022-00526-x
