# Supplementary material for: Psychosocial interventions for depression among young people in Sub-Saharan Africa: a systematic review and meta-analysis
Source: Int J Ment Health Syst. 2024 Jun 22;18:24. doi: 10.1186/s13033-024-00642-w (PMC11193191; doi:10.1186/s13033-024-00642-w)
Supplement: Supplementary file 3 — Supplementary material 3. Search strategy. [file 13033_2024_642_MOESM3_ESM.docx]

**Additional File 3: Search Strategy (9/05/2024)**

**1a. Medline (OVID)**

| **Sequence** | **Search Terms** | **Hits** |
| --- | --- | --- |
| 1 | exp Adolescent | 2245124 |
| 2 | (Adolescent* or Teenager* or Teen* or youth* or young person* or young people or youngster* or young adult* or student* or high school* or college* or secondary school*).mp. | 3292595 |
| 3 | 1 or 2 | 3292595 |
| 4 | exp "Africa South of the Sahara"/ | 268712 |
| 5 | (((Sub-Saharan Africa or Africa* or West* Africa or East* Africa or South* Africa or Central Africa or Angola or Benin or Botswana or Burkina Faso or Burundi or Cape Verde or Cameroon or Central African Republic or Chad or Comoros or Democratic Republic of Congo or Congo or Cote d'Ivoire or Equatorial Guinea or Eritrea or Eswatini or Ethiopia or Gabon or Gambia or Guinea or Guinea-Bissau or Kenya or Lesotho or Liberia or Madagascar or Malawi or Mali or Mauritania or Mauritius or Mozambique or Namibia or Niger or Nigeria or Rwanda or Sao Tome) and Principe) or Senegal or Seychelles or Sierra Leone or Somalia or South Africa or South Sudan or Sudan or Tanzania or Togo or Uganda or Zambia or Zimbabwe).mp. | 150640 |
| 6 | 4 or 5 | 306620 |
| 7 | exp Psychotherapy/ | 223479 |
| 8 | (psychosocial* or psychotherap* or psychological or psychoeducation or behavio?ral therapy or cognitive behavio?ral therapy or CBT or interpersonal therapy or IPT, or problem-solving therapy or counsel* or narrative therapy).mp. | 1029573 |
| 9 | 7 or 8 | 1111844 |
| 10 | Depression/ | 157513 |
| 11 | exp Depressive Disorder/ | 124685 |
| 12 | (depression or depressive disorder* or dysthymia or depressive symptom*).mp. | 558693 |
| 13 | 10 or 11 or 12 | 559500 |
| 14 | 3 and 6 and 9 and 13 | 946 |
| 15 | (intervention* or Treat* or therap* or trial* or project* or program*).mp. | 1345313 |
| 16 | 14 and 15 | **623** |

**1b. PsycINFO**

| **Sequence** | **Search Terms** | **Hits** |
| --- | --- | --- |
| 1 | (Adolescent* or Teenager* or Teen* or youth* or young person* or young people or youngster* or young adult* or student* or high school* or college* or secondary school).mp. | 1524919 |
| 2 | (((Sub-Saharan Africa or SubSaharan Africa or Africa* or West* Africa or East* Africa or South* Africa or Central Africa or Angola or Benin or Botswana or Burkina Faso or Burundi or Cape Verde or Cameroon or Central African Republic or Chad or Comoros or Democratic Republic of Congo or Congo or Cote d'Ivoire or Equatorial Guinea or Eritrea or Eswatini or Ethiopia or Gabon or Gambia or Guinea or Guinea-Bissau or Kenya or Lesotho or Liberia or Madagascar or Malawi or Mali or Mauritania or Mauritius or Mozambique or Namibia or Niger or Nigeria or Rwanda or Sao Tome) and Principe) or Senegal or Seychelles or Sierra Leone or Somalia or South Africa or South Sudan or Sudan or Tanzania or Togo or Uganda or Zambia or Zimbabwe).mp. | 119221 |
| 3 | exp psychotherapy/ | 147192 |
| 4 | (psychosocial* or psychotherap* or psychological or psychoeducation or behavio?ral therapy or cognitive behavio?ral therapy or CBT or interpersonal therapy or IPT, or problem-solving therapy or counsel* or narrative therapy).mp. | 1166478 |
| 5 | 3 or 4 | 1166478 |
| 6 | exp "depression (emotion)"/ | 20609 |
| 7 | exp major depression/ | 147890 |
| 8 | (depression or depressive disorder* or dysthymia or depressive symptom*).mp. | 370492 |
| 9 | 6 or 7 or 8 | 370492 |
| 10 | 1 and 2 and 5 and 9 | 311 |
| 11 | (intervention* or Treat* or therap* or trial* or project* or program*).mp. | 1760009 |
| 12 | 10 and 11 | **234** |

**1c. Web of Science**

| **Sequence** | **Search Terms** |
| --- | --- |
| **1** | Adolescent* or Teenager* or Teen* or youth* or "young person*" or "young people" or youngster* or student* or "high school*" or college* or "secondary school*" |
| **2** | Sub$Saharan Africa or "Sub Saharan Africa" or "West* Africa" or "East* Africa" or "South* Africa" or "Central Africa" or Angola or Benin or Botswana or "Burkina Faso" or Burundi or "Cape Verde" or Cameroon or "Central African Republic" or Chad or Comoros or "Democratic Republic of Congo" or Congo or "Cote d'Ivoire" or "Equatorial Guinea" or Eritrea or Eswatini or Ethiopia or Gabon or Gambia or Guinea or Guinea-Bissau or Kenya or Lesotho or Liberia or Madagascar or Malawi or Mali or Mauritania or Mauritius or Mozambique or Namibia or Niger or Nigeria or Rwanda or "Sao Tome and Principe" or Senegal or Seychelles or "Sierra Leone" or Somalia or "South Africa" or "South Sudan" or Sudan or Tanzania or Togo or Uganda or Zambia or Zimbabwe |
| **3** | psychosocial* or psychotherap* or psychological or psychoeducation or "behavio?ral therapy" or "cognitive behavio?ral therapy" or CBT or "interpersonal therapy" or IPT or "problem-solving therapy" or counsel* or "narrative therapy" |
| **4** | depression or "depressive disorder*" or dysthymia or "depressive symptom"* |
| **5** | intervention* or Treat* or therap* or trial* or project* or program* |
|  | 1 AND 2 AND 3 AND 4 AND 5 = **372** |

**1d. COCHRANE (CENTRAL)**

| **Sequence** | **Search Terms** | **Hits** |
| --- | --- | --- |
| 1 | MeSH descriptor: [Adolescent] | 165356 |
| 2 | Adolescent* or Teenager* or Teen* or youth* or young person* or young people or youngster* or young adult* or student* or high school* or college* or secondary school* | 404172 |
| 3 | 1 or 2 | 404172 |
| 4 | MeSH descriptor: [Africa South of the Sahara] | 2636 |
| 5 | Sub-Saharan Africa or Subsaharan Africa or Sub Saharan Africa or West* Africa or East* Africa or South* Africa or Central Africa or Angola or Benin or Botswana or Burkina Faso or Burundi or Cape Verde or Cameroon or Central African Republic or Chad or Comoros or Democratic Republic of Congo or Congo or Cote d'Ivoire or Equatorial Guinea or Eritrea or Eswatini or Ethiopia or Gabon or Gambia or Guinea or Guinea-Bissau or Kenya or Lesotho or Liberia or Madagascar or Malawi or Mali or Mauritania or Mauritius or Mozambique or Namibia or Niger or Nigeria or Rwanda or Sao Tome and Principe or Senegal or Seychelles or Sierra Leone or Somalia or South Africa or South Sudan or Sudan or Tanzania or Togo or Uganda or Zambia or Zimbabwe | 30904 |
| 6 | 4 or 5 | 30904 |
| 7 | MeSH descriptor: [Psychotherapy] | 19864 |
| 8 | psychosocial* or psychotherap* or psychological or psychoeducation or behavio?ral therapy or cognitive behavio?ral therapy or CBT or interpersonal therapy or IPT or problem-solving therapy or counsel* or narrative therapy | 173570 |
| 9 | 7 or 8 | 173570 |
| 10 | MeSH descriptor: [Depression] | 107435 |
| 11 | depression or depressive disorder* or dysthymia or depressive symptom* | 113383 |
| 12 | 10 or 11 | 113384 |
| 13 | 3 and 6 and 9 and 12 | 861 |
| **14** | intervention* or Treat* or therap* or trial* or project* or program* | 2161339 |
| **15** | 13 and 14 (+ Limit to Trials) | **409** |
