# Supplementary material for: Psychosocial interventions for depression among young people in Sub-Saharan Africa: a systematic review and meta-analysis
Source: Int J Ment Health Syst. 2024 Jun 22;18:24. doi: 10.1186/s13033-024-00642-w (PMC11193191; doi:10.1186/s13033-024-00642-w)
Supplement: Supplementary file 4 — Supplementary material 4. Reasons for exclusion. [file 13033_2024_642_MOESM4_ESM.docx]

# **Additional File 4: Reasons for Exclusion**

| **References** | **Titles** | **Reasons for Exclusion** |
| --- | --- | --- |
| Abas et al*.* (2020) | The effect of co-morbid anxiety on remission from depression for people participating in a randomized controlled trial of the Friendship Bench intervention in Zimbabwe | Wrong population. |
| Dorsey *et al.* (2020) | Effectiveness of Task-Shifted Trauma-Focused Cognitive Behavioral Therapy for Children Who Experienced Parental Death and Posttraumatic Stress in Kenya and Tanzania: A Randomized Clinical Trial | Wrong outcome. Wrong population |
| Ede et al*.* (2022) | The Effect of Rational Emotive Behaviour Therapy on Post-Traumatic Depression in Flood Victims | Wrong population |
| Gureje, O., et al. (2015). | A cluster randomized clinical trial of a stepped care intervention for depression in primary care (STEPCARE)--study protocol. | Wrong population |
| Mon, M. M., et al. (2016) | Effectiveness of Mindfulness Intervention on Psychological Behaviors Among Adolescents With Parental HIV Infection: A Group-Randomized Controlled Trial | Wrong outcome. |
| Salihu, D., et al. (2021) | Effects of an African Circle Dance Programme on Internally Displaced Persons with Depressive Symptoms: a Quasi-Experimental Study. | Wrong population |
| Senyonyi, R. M. (2013). | CBT group counselling intervention for HIV transmission risk behavior in perinatally infected adolescents | Wrong outcome |
| Woollett, N., et al. (2020) | Trauma-informed art and play therapy: Pilot study outcomes for children and mothers in domestic violence shelters in the United States and South Africa | Wrong population |
| Zuilkowski, S. S., et al. (2016). | Youth and resilience in post conflict settings: An intervention for war-affected youth in Sierra Leone. | Wrong outcome |
| Bantjes et al. (2021) | A Web-Based Group Cognitive Behavioral Therapy Intervention for Symptoms of Anxiety and Depression Among University Students: Open-Label, Pragmatic Trial | Wrong population |
| Ertl, V., et al. (2011) | Community-Implemented Trauma Therapy for Former Child Soldiers in Northern Uganda: A Randomized Controlled Trial. | Wrong outcome. |
| Im, H. and L. E. T. Swan (2022) | Factors Influencing Improvement of Trauma-Related Symptoms Among Somali Refugee Youth in Urban Kenya. | Wrong outcome. |
| Rossouw, J., et al. (2016). | A pilot and feasibility randomised controlled study of Prolonged Exposure Treatment and supportive counselling for post-traumatic stress disorder in adolescents: a third world, task-shifting, community-based sample. | Wrong outcome |
| Kane, J. C., et al. (2016). | Moderators of treatment response to trauma-focused cognitive behavioral therapy among youth in Zambia. | Wrong outcome |
| Jordans, M. J. D., et al. (2013) | Treatment Processes of Counselling for Children in South Sudan: A Multiple n=1 Design | Wrong outcome |
| O'Donnell, K., et al. (2014) | Treating Maladaptive Grief and Posttraumatic Stress Symptoms in Orphaned Children in Tanzania: Group-Based Trauma-Focused Cognitive-Behavioral Therapy | Wrong population |
| Olowokere and Okanlawon (2018). | Improving vulnerable school children's psychosocial health outcomes through resilience-based training and peer-support activities: a comparative prospective study. | Wrong outcome |
| Simms, V., et al. (2022). | Peer-led counselling with problem discussion therapy for adolescents living with HIV in Zimbabwe: A cluster-randomised trial. | Wrong outcome |
| Tol, W. A., et al. (2020). | Guided self-help to reduce psychological distress in South Sudanese female refugees in Uganda: a cluster randomised trial. | Wrong outcome |
| Ugwu, G. C., et al. (2022). | Efficacy of Rational Emotive Behavior Therapy on Depression Among Children with Learning Disabilities: Implications for Evaluation in Science Teaching. | Wrong population |
| Abas *et al.* (2020) | The effect of co-morbid anxiety on remission from depression for people participating in a randomised controlled trial of the Friendship Bench intervention in Zimbabwe | Wrong population. |
| Dorsey *et al.* (2020) | Effectiveness of Task-Shifted Trauma-Focused Cognitive Behavioural Therapy for Children Who Experienced Parental Death and Posttraumatic Stress in Kenya and Tanzania: A Randomized Clinical Trial | Wrong outcome. Wrong population |
| Ede *et al.* (2022) | The Effect of Rational Emotive Behaviour Therapy on Post-Traumatic Depression in Flood Victims | Wrong population |
| Gureje, O., et al. (2015). | A cluster randomized clinical trial of a stepped care intervention for depression in primary care (STEPCARE)--study protocol. | Wrong population |
| Mon, M. M., et al. (2016) | Effectiveness of Mindfulness Intervention on Psychological Behaviours Among Adolescents With Parental HIV Infection: A Group-Randomized Controlled Trial | Wrong outcome. |
| Salihu, D., et al. (2021) | Effects of an African Circle Dance Programme on Internally Displaced Persons with Depressive Symptoms: a Quasi-Experimental Study. | Wrong population |
| Senyonyi, R. M. (2013). | CBT group counselling intervention for HIV transmission risk behaviour in perinatally infected adolescents | Wrong outcome |
| Woollett, N., et al. (2020) | Trauma-informed art and play therapy: Pilot study outcomes for children and mothers in domestic violence shelters in the United States and South Africa | Wrong population |
| Zuilkowski, S. S., et al. (2016). | Youth and resilience in post conflict settings: An intervention for war-affected youth in Sierra Leone. | Wrong outcome |
| Bantjes et al. (2021) | A Web-Based Group Cognitive Behavioural Therapy Intervention for Symptoms of Anxiety and Depression Among University Students: Open-Label, Pragmatic Trial | Wrong population |
| Bantjes et al. (2024) | Comparative effectiveness of remote digital gamified and group CBT skills training interventions for anxiety and depression among college students: Results of a three-arm randomised controlled trial. | Wrong outcome measure |
| Ertl, V., et al. (2011) | Community-Implemented Trauma Therapy for Former Child Soldiers in Northern Uganda: A Randomized Controlled Trial. | Wrong outcome. |
| Im, H. and L. E. T. Swan (2022) | Factors Influencing Improvement of Trauma-Related Symptoms Among Somali Refugee Youth in Urban Kenya. | Wrong outcome. |
| Rossouw, J., et al. (2016). | A pilot and feasibility randomised controlled study of Prolonged Exposure Treatment and supportive counselling for post-traumatic stress disorder in adolescents: a third world, task-shifting, community-based sample. | Wrong outcome |
| Kane, J. C., et al. (2016). | Moderators of treatment response to trauma-focused cognitive behavioural therapy among youth in Zambia. | Wrong outcome |
| Jordans, M. J. D., et al. (2013) | Treatment Processes of Counselling for Children in South Sudan: A Multiple n=1 Design | Wrong outcome |
| O'Donnell, K., et al. (2014) | Treating Maladaptive Grief and Posttraumatic Stress Symptoms in Orphaned Children in Tanzania: Group-Based Trauma-Focused Cognitive-Behavioral Therapy | Wrong population |
| Olowokere and Okanlawon (2018). | Improving vulnerable school children's psychosocial health outcomes through resilience-based training and peer-support activities: a comparative prospective study. | Wrong outcome |
| Simms, V., et al. (2022). | Peer-led counselling with problem discussion therapy for adolescents living with HIV in Zimbabwe: A cluster-randomised trial. | Wrong outcome |
| Tol, W. A., et al. (2020). | Guided self-help to reduce psychological distress in South Sudanese female refugees in Uganda: a cluster randomised trial. | Wrong outcome |
| Ugwu, G. C., et al. (2022). | Efficacy of Rational Emotive Behaviour Therapy on Depression Among Children with Learning Disabilities: Implications for Evaluation in Science Teaching. | Wrong population |
| Yi et al. (2024) | Cognitive behavior, art, and music therapies intervention for treating the depression of children: A randomized control trial. | Wrong population |
| Sorsdahl et al. (2024) | Project ASPIRE: A feasibility randomized controlled trial of a brief intervention for reducing risk of depression and alcohol-related harms among South African adolescents. | Wrong outcome |
| Byansi *et al.*, 2022 | The Short-Term Impact of a Combination Intervention on Depressive Symptoms Among School-Going Adolescent Girls in Southwestern Uganda: The Suubi4Her Cluster Randomized Trial. | Wrong Intervention |
| Donenberg *et al.,* 2023 | Results of the Kigali Imbereheza Project: A 2-Arm Individually Randomized Trial of TI-CBT Enhanced to Address ART Adherence and Mental Health for Rwandan Youth Living With HIV | Wrong  Outcome |
